# A brief morning rest period benefits cardiac repair in pressure overload hypertrophy and postmyocardial infarction

**DOI:** 10.1172/jci.insight.164700

**Published:** 2022-11-22

**Authors:** Cristine J. Reitz, Mina Rasouli, Faisal J. Alibhai, Tarak N. Khatua, W. Glen Pyle, Tami A. Martino

**Affiliations:** Department of Biomedical Sciences, University of Guelph, Guelph, Ontario Canada.

**Keywords:** Cardiology, Cardiovascular disease

## Abstract

Rest has long been considered beneficial to patient healing; however, remarkably, there are no evidence-based experimental models determining how it benefits disease outcomes. Here, we created an experimental rest model in mice that briefly extends the morning rest period. We found in 2 major cardiovascular disease conditions (cardiac hypertrophy, myocardial infarction) that imposing a short, extended period of morning rest each day limited cardiac remodeling compared with controls. Mechanistically, rest mitigates autonomic-mediated hemodynamic stress on the cardiovascular system, relaxes myofilament contractility, and attenuates cardiac remodeling genes, consistent with the benefits on cardiac structure and function. These same rest-responsive gene pathways underlie the pathophysiology of many major human cardiovascular conditions, as demonstrated by interrogating open-source transcriptomic data; thus, patients with other conditions may also benefit from a morning rest period in a similar manner. Our findings implicate rest as a key driver of physiology, creating a potentially new field — as broad and important as diet, sleep, or exercise — and provide a strong rationale for investigation of rest-based therapy for major clinical diseases.

## Graphical abstract

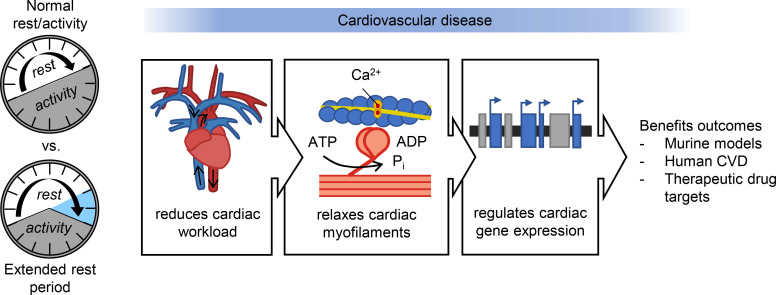

## Introduction

Cardiovascular disease is a major health care and economic burden, with complex clinical phenotypes that frequently progress toward heart failure, for which there is no cure (1). Circadian rhythms of daily rest and activity contribute to the pathogenesis and pathophysiology of cardiovascular disease (2, 3). Historically, physicians have long aligned with the notion that rest benefits patient outcomes (4). Indeed Hippocrates, the father of medicine, is quoted as saying “rest as soon as there is pain.” However, clinical recommendations have irrationally tended toward absolute bed rest, resulting in a conundrum as studies infer no benefits or even worse outcomes with complete immobilization in bed (5). Clinical studies also lack clarity as they focus only on the negative consequences of nocturnal disruption on the heart, for example due to insufficient sleep (6–9), social jet lag (10), or shift work (11). Similarly, experimental rodent studies also only focus on the adverse effects of environmental desynchrony on cardiac repair (reviewed in refs. 12–14), and thus fail to recapitulate the full range of benefits of rest on cardiovascular healing. Indeed, there is little definitive evidence supporting how or why rest benefits heart health and repair. As a result, fundamental physiological and molecular mechanisms of rest remain unclear, and there are no evidence-based data available to benefit individuals with cardiovascular disease. This experimental study adds a short period of rest at the start of the animal’s time awake (i.e., recapitulating what would be early morning in humans), in addition to the normal nighttime rest period, to demonstrate how rest drives physiological and molecular responses to benefit cardiovascular disease outcomes.

## Results

### An extended-rest mouse model protects against cardiovascular disease.

**Figure 1:**
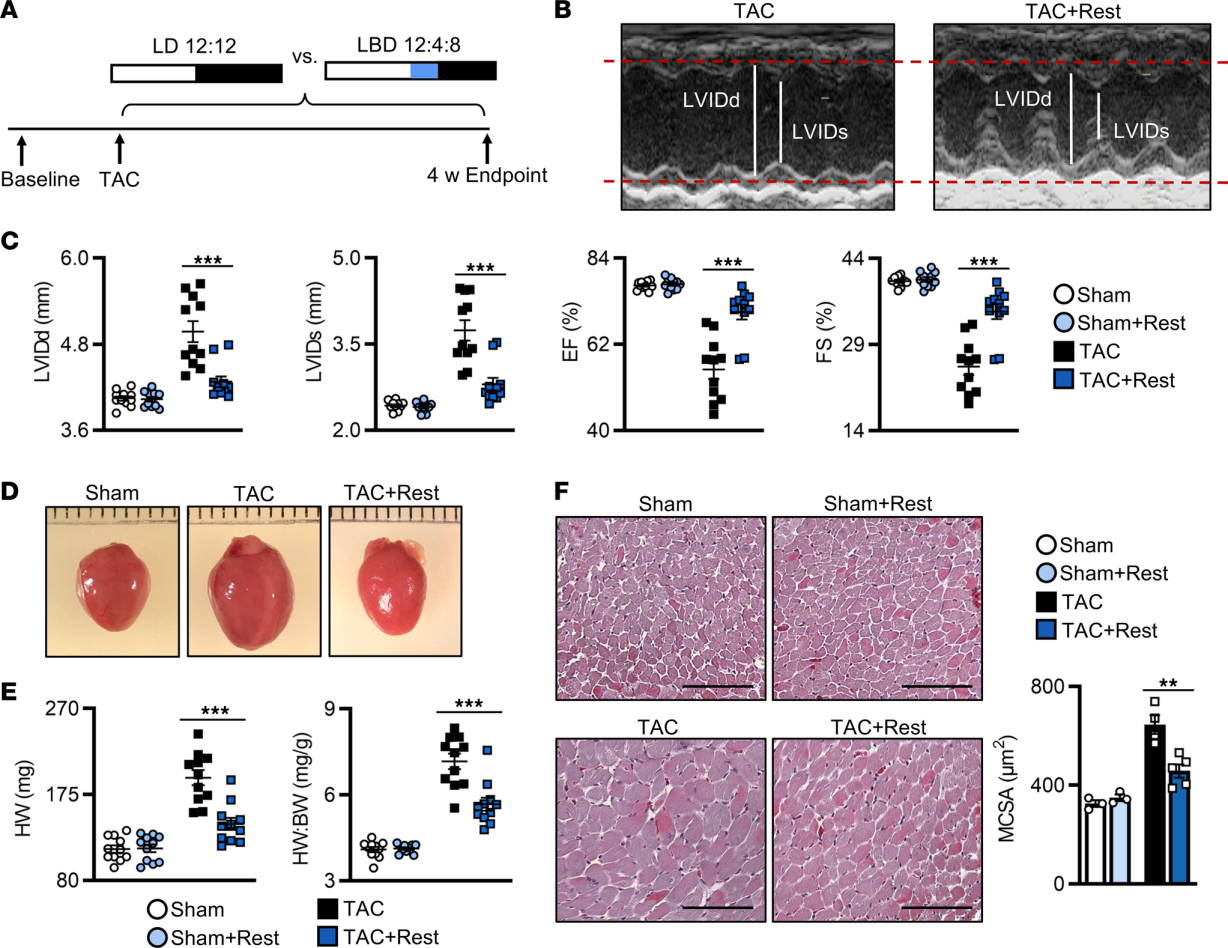
Rest benefits outcomes in pressure overload–induced cardiac hypertrophy (TAC model). (**A**) Rest model and cardiac hypertrophy experimental design: mice underwent baseline echocardiography followed by transverse aortic constriction (TAC) and were randomized to control conditions (12 hour-light/12-hour dark cycle; LD 12:12) versus the rest model (12-hour light/4-hour blue light/8-hour dark; LBD 12:4:8) for up to 4 weeks. (**B**) Representative M-mode echocardiography at 4 weeks after TAC, showing (**C**) smaller left ventricular internal dimensions at diastole (LVIDd) and systole (LVIDs) and better % ejection fraction (EF) and fractional shortening (FS) in mice under the rest model. *n* = 11 mice/group. ***P < 0.001, unpaired, 2-tailed Student’s *t* test. (**D**) Representative images and (**E**) quantification of heart weight (HW) and HW/BW at 4 weeks after TAC. *n* = 11 mice/group. ***P < 0.001, unpaired Student’s *t* test. (**F**) Representative images and quantification of cardiomyocyte cross-sectional area at 4 weeks after TAC. *n* = 3 sham hearts/group, *n* = 5 TAC hearts/group. **P < 0.01, unpaired Student’s *t* test. Scale bar: 100 μm.

Rest is a fundamental aspect of human biology, yet little is known about the biological effects of adding a brief daily rest period on healing of peripheral organs such as the heart. To determine this, male C57BL/6N WT mice were subjected to two heart disease models, each recapitulating a major human cardiovascular disease (cardiac hypertrophy, myocardial infarction [MI]), followed by imposition of a short extended daily rest period, and benefits were observed in vivo. We first examined pathological cardiac hypertrophy using the murine transverse aortic constriction (TAC) model. After recovery from surgery, mice were randomized to either (a) a normal 12-hour light (L)/12-hour dark (D) cycle (normal model; LD 12:12; control) or (b) 12 hours of light plus an additional 4 hours of blue (B) light (420–520 nm) to stimulate the retinal photoreceptors to delay the onset of activity (15), followed by 8 hours of dark (rest model; LBD 12:4:8; + rest). Animals were maintained under these conditions for 4 weeks after surgery, which recapitulates the cardiac hypertrophy phenotype in this model (Figure 1A). As expected, the TAC mice developed cardiac hypertrophy, consistent with previous studies (16–18). In contrast, we found that mice provided additional daily rest after TAC (TAC+Rest) developed less adverse cardiac remodeling and maintained better cardiac function, as shown by echocardiography (Figure 1B), with significantly smaller left ventricular internal dimensions at diastole (LVIDd) and systole (LVIDs) and better ejection fraction (% EF) and fractional shortening (% FS) by 4 weeks in vivo (Figure 1C and Supplemental Table 1; supplemental material available online with this article; https://doi.org/10.1172/jci.insight.164700DS1). The extended rest period also protected against the development of cardiac hypertrophy (Figure 1D), as evidenced by significantly smaller heart weight (HW) and HW/body weight (BW) ratios compared with controls (Figure 1E). At the cellular level, significantly less cardiomyocyte hypertrophy developed in the TAC+Rest hearts, with smaller cardiomyocyte cross-sectional area, as compared with controls by histopathology (Figure 1F). Collectively, these data validated that a short additional rest period reduced adverse cardiac remodeling, suggesting a feasible approach to limit disease severity.

**Figure 2:**
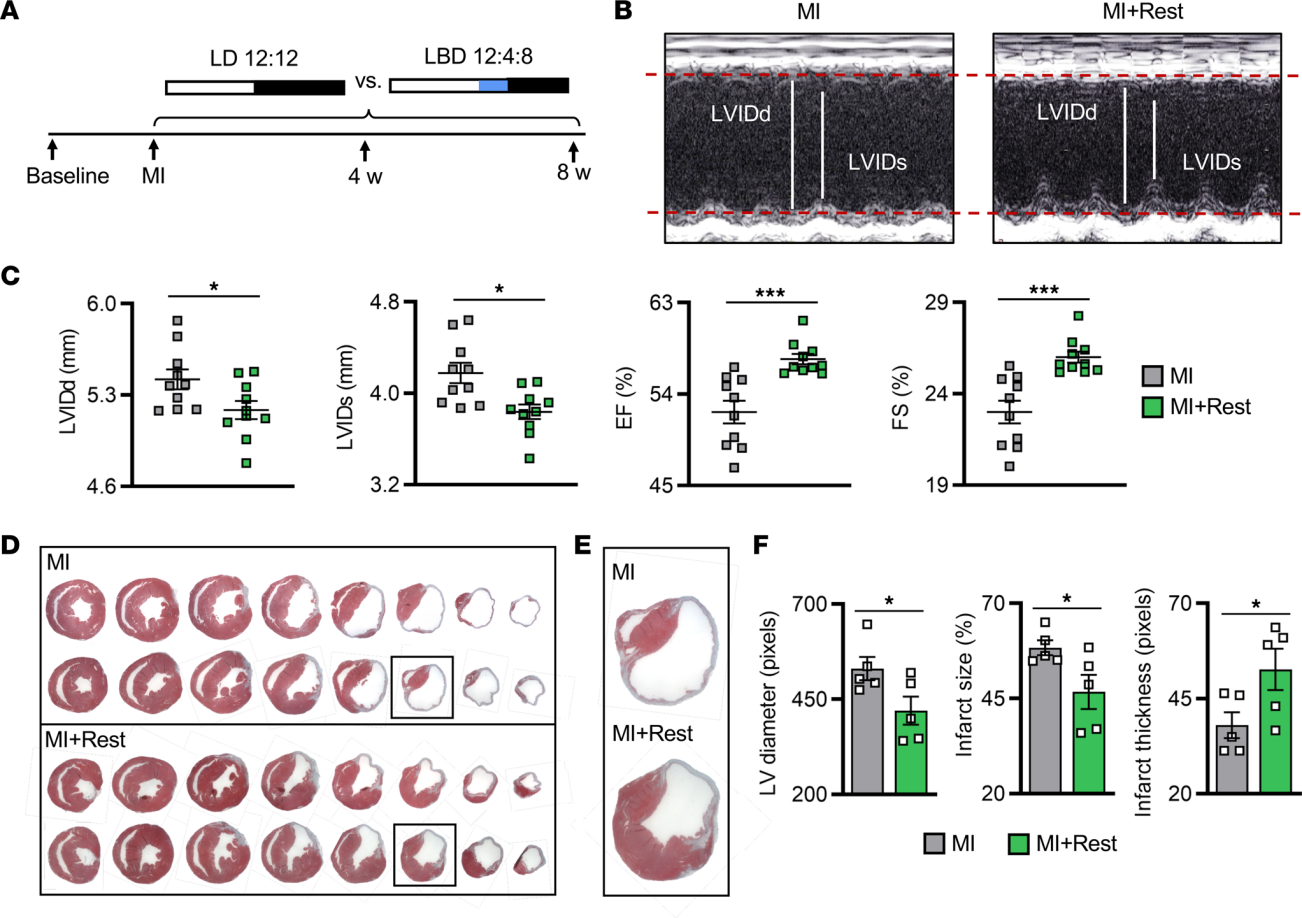
Rest benefits outcomes in ischemic heart disease. (**A**) Rest model and myocardial infarction (MI) experimental design. (**B**) Representative M-mode echocardiography at 8 weeks after MI, showing (**C**) smaller LVIDd and LVIDs and better % EF and % FS in mice under the rest model. *n* = 10 mice/group. (**D**) Representative histopathology, (**E**) close-up of infarct region, and (**F**) quantification of LV diameter and infarction expansion and integrity in MI+Rest hearts at 8 weeks after MI. *n* = 5 mice/group. *P < 0.05 and ***P < 0.001, unpaired Student’s *t* test.

We then used a second model to investigate whether rest can benefit repair in ischemic heart disease, this time using MI, mimicking the leading cause of death in humans. Mice were subjected to permanent left anterior descending coronary artery ligation and immediately randomized to the same treatment groups, either control (normal LD 12:12) or the rest model (LBD 12:4:8; +Rest). Animals were maintained for up to 8 weeks after MI, recapitulating the progression to heart failure (Figure 2A). As expected, the MI mice developed adverse cardiac remodeling, consistent with our previous studies (19–21). In contrast, the MI+Rest mice developed substantially less left ventricular dilation (Figure 2B), and longitudinal echocardiographic evaluation revealed better-preserved cardiac function by 8 weeks after MI (Figure 2C and Supplemental Table 2). The extended rest period also protected against infarct expansion, resulting in smaller scars and better infarct thickness and integrity, as demonstrated by histopathology at 8 weeks after MI (Figure 2, D–F). Collectively, using two models of acquired heart disease, these studies demonstrated that an additional brief period of rest at the start of the animal’s time awake, as a model of early morning in humans, limited adverse cardiac remodeling and improved long-term outcomes.

### Light-induced manipulation of the circadian system to impose rest.

The data thus far suggest that briefly extending the daily rest period profoundly preserved cardiac structure and function in two murine models that mirror the clinical features of human cardiovascular disease. Taking advantage of light as the primary zeitgeber regulating daily rest and activity rhythms, we next evaluated the effects of spectral distribution. In the mammalian retina, intrinsically photosensitive melanopsin-expressing retinal ganglionic cells (ipRGCs) are responsible for the synchronizing effects of external light on the endogenous circadian timing system in the brain. These ipRGCs, as well as their axonal projections to the hypothalamic suprachiasmatic nucleus, have been described in rodents (15, 22–24) and humans (25, 26), with a similar maximal sensitivity to approximately 480 nm short-wavelength light (e.g., blue) but not long-wavelength (e.g., red) light. The sensitivity of this system to light for imposing rest in murine heart disease is unknown.

**Figure 3:**
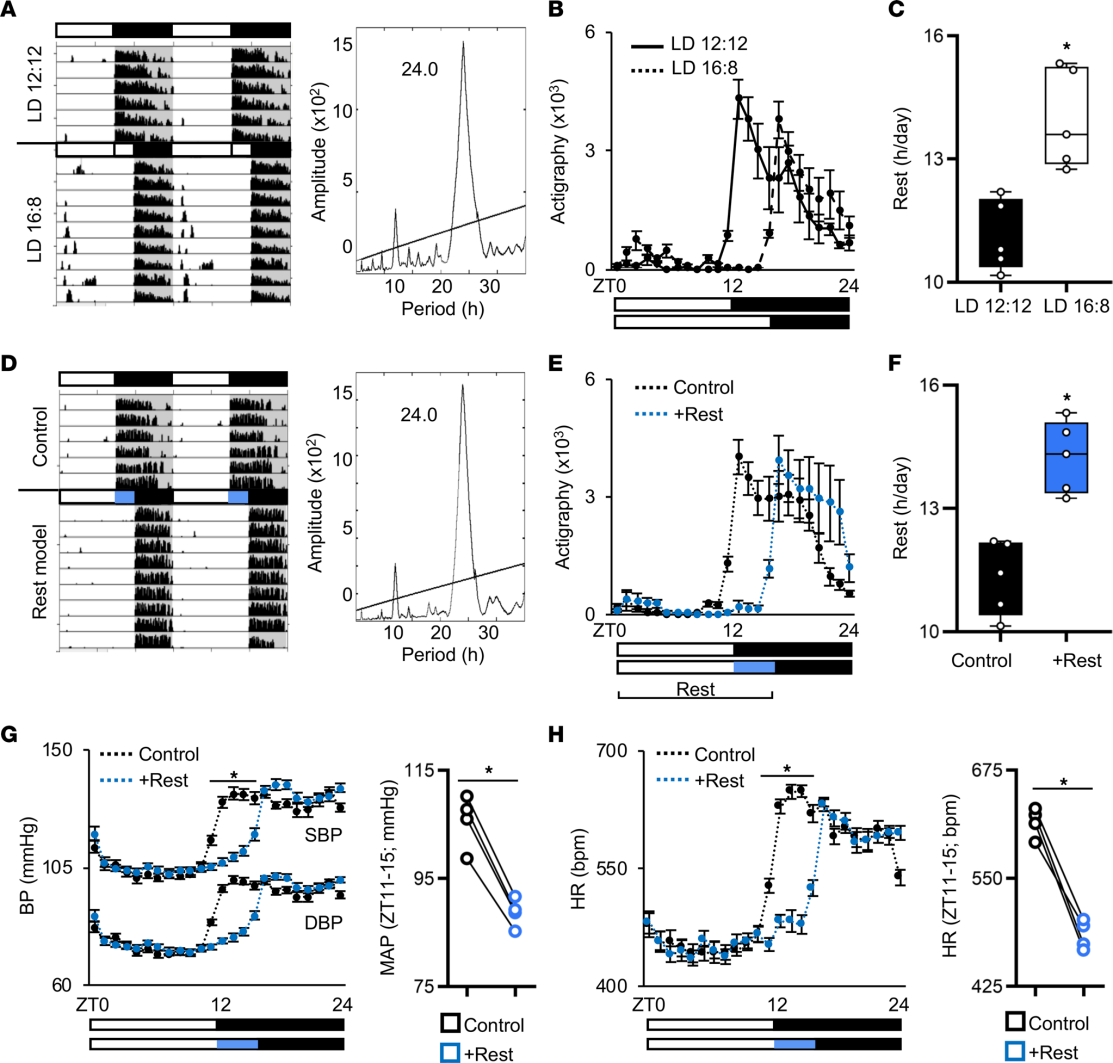
Diurnal rest-activity rhythms and cardiovascular hemodynamics in a mouse model of rest. (**A**) Representative actigraphy and periodogram, (**B**) activity quantification, and (**C**) hours of rest per day in mice under normal LD 12:12 versus extended light (LD 16:8). *n* = 5 mice/group. (**D**) Representative actigraphy and periodogram, (**E**) activity quantification, and (**F**) hours of rest per day in mice under the rest model (+Rest) versus control. *n* = 5 mice/group. Additional actigraphs under LD and constant darkness (DD) are provided in Supplemental Figures 1 and 2. (**G**) Diurnal rhythms in systolic and diastolic blood pressure (SBP and DBP) and quantification of average mean arterial pressure (MAP) from zeitgeber time (ZT) 11–15 and (**H**) diurnal heart rate in healthy mice under rest model versus control. *n* = 4 mice/group. *P < 0.001, paired Student’s *t* test.

To characterize the rest model, we used running-wheel actigraphy and found that an additional 4 hours of normal broad spectrum white light during what would otherwise be the start of the active period resulted in a 4-hour extended rest period (Figure 3, A and B). Importantly, the animals still maintained a normal circadian period of approximately 24 hours (Figure 3A), even though the number of hours per day spent at rest were significantly increased (Figure 3C). In contrast, mice exposed to red light (>620 nm) showed no change in daily activity patterns, as expected (Supplemental Figure 1, A and B). Next, we determined that short-wavelength blue light alone (420–520 nm, activates ipRGCs) was sufficient to delay activity onset while maintaining a normal approximately 24-hour circadian period (Figure 3D), thus imposing more hours of rest (Figure 3, E and F). Notably, even though the animals spent more time at rest, there was no difference in their relative level of activity during time spent awake (Supplemental Figure 1, C–F). We also assessed the impact of the additional rest period on endogenous rhythms and found that both whole body behavioral rhythms and circadian clock gene cycling in the heart align with the rest period, consistent with the notion that endogenous rhythms continue to cycle in phase with the external environment (Supplemental Figure 2). Importantly, we then investigated cardiac hemodynamics in our control and rest mice by *in vivo* radiotelemetry. We found reduced hemodynamic load during the extended rest period, with significantly lower systolic and diastolic blood pressure (SBP and DBP) and lower cardiac afterload (mean arterial pressure; MAP) (Figure 3G). Heart rate was also significantly reduced under the rest model compared with controls (Figure 3H). Our mammalian physiology undergoes profound daily rhythms that are important for cardiovascular health (27–29) and consistent with this, a short period of additional morning rest can benefit cardiac physiology.

### Rest induced contractile function and phosphorylation of sarcomeric myofilaments and precision remodeling in a cardiac disease–specific manner.

The cardiac sarcomere is the basic contractile unit of the heart, and sarcomeric dysfunction drives changes in cardiac muscle contractility central to impaired cardiac function, as reviewed in a previous study (30). The structure and function of the cardiac sarcomere is regulated by phosphorylation of the myofilament proteins (31). In heart failure, altered myofilament phosphorylation is a key driver of contractile dysfunction and disease progression (32). As a result, targeting the posttranslational modification of cardiac myofilaments has become a new frontier in improving cardiac function in heart failure (33). Our model may provide a potentially novel and promising nonpharmaceutical approach, but the effects of briefly extending morning rest on contractility parameters of the remodeling heart are unknown.

**Figure 4:**
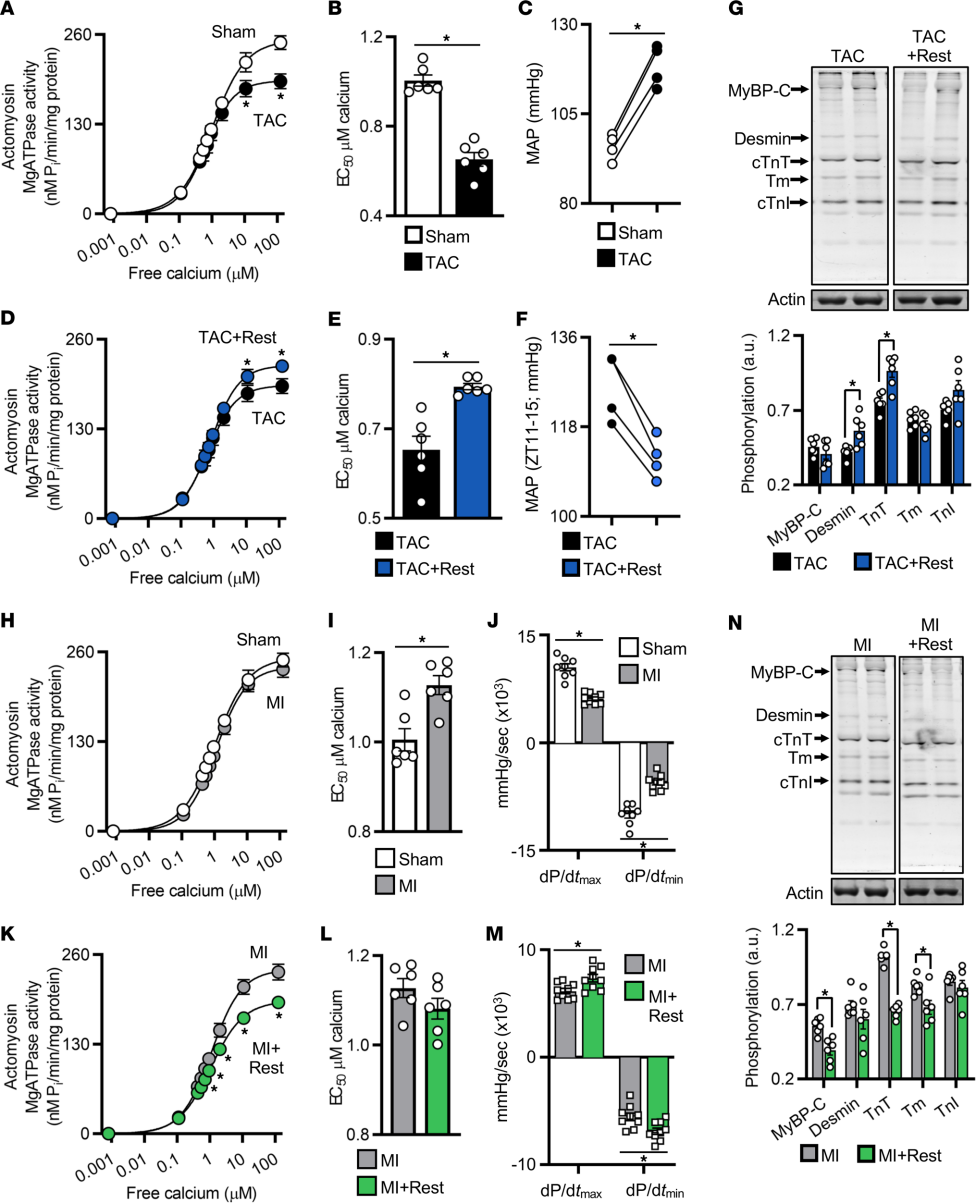
Rest reduces cardiac workload through intrinsic effects on the cardiac myofilaments. (**A**) Cardiac actomyosin MgATPase activity and (**B**) EC_50_ were reduced at 4 weeks after TAC, (**C**) consistent with pathological cardiac afterload (mean arterial pressure; MAP) in the TAC model (additional cardiac hemodynamics are provided in Supplemental Figure 3). (**D**) Myofilament activity and (**E**) EC_50_ and (**F**) MAP were normalized in TAC+Rest hearts, consistent with (**G**) increased myofilament protein phosphorylation. TAC data from **A** and **B** are repeated in **D** and **E** for comparison. (**H**) Cardiac actomyosin MgATPase activity was maintained at 4 weeks after MI, (**I**) with increased EC_50_, (**J**) consistent with reduced in vivo cardiac contractility. (**K**) Extended rest lowered myofilament ATP consumption (**L**) but not EC_50_ (**M**) consistent with better in vivo cardiac contractility and (**N**) reduced myofilament protein phosphorylation. MI data are repeated in **H** and **I** and in **K** and **L** for comparison. Murine heart tissue collected at ZT15, 4 weeks after surgery. *n* = 6/group for ATPase and phosphorylation, *n* = 4/group for in vivo radiotelemetry, *n* = 9/group for in vivo pressure-volume hemodynamics. For MgATPase data, *P < 0.005; for all other data, *P < 0.05, by unpaired Student’s *t* test.

To test for sarcomeric relevance, we first determined how myofilament function is altered from baseline in pathological cardiac hypertrophy using TAC hearts examined by actomyosin MgATPase activity assay (34). As compared with healthy hearts under normal light/dark conditions, the TAC hearts had significantly reduced maximal MgATPase activity (Figure 4A), and EC_50_ was similarly decreased (Figure 4B), suggestive of impaired sarcomere activity at high Ca^2+^ concentrations. These findings coincided with the increased cardiac afterload in the TAC hearts (e.g., MAP) (Figure 4C). In contrast, in order to determine whether rest changes cardiac myofilament function after pressure overload, we assessed TAC+Rest hearts versus the TAC controls above and found that with the brief additional period of rest, TAC+Rest hearts had better maximal MgATPase activity (Figure 4D); normalized EC_50_ (Figure 4E); and reduced cardiac afterload (Figure 4F), SBP, DBP, and heart rate (Supplemental Figure 3, A and B), where values were maintained similar to sham controls. Strikingly, TAC+Rest hearts had significantly higher phosphorylation levels of desmin and troponin T (TnT) (Figure 4G), providing a biological basis underlying reduced stress on the heart. Collectively, these observations are consistent with the notion that the brief morning period of rest offsets pathological myocardial remodeling by preserving contractile function at the level of the cardiac sarcomere.

We next investigated how rest influences sarcomere function in remodeling myocardium after MI. First, we assessed changes in myofilament function from baseline in the MI model and found that, as compared with healthy hearts under a normal light/dark cycle, the MI hearts maintained normal actomyosin MgATPase activity at all levels of calcium (Figure 4H); however, we did note that EC_50_ was increased (Figure 4I), suggestive of a change in myofilament calcium sensitivity and coinciding with decreased cardiac contractility as demonstrated by reduced dP/d*t*_max_ and dP/d*t*_min_ values (Figure 4J). In contrast, to determine the effects of rest on cardiac myofilaments after MI, we assessed MI+Rest hearts versus MI controls and found that with the brief additional period of rest, MI+Rest hearts had reduced maximal MgATPase activity (Figure 4K) and no change in EC_50_ (Figure 4L), suggestive of reduced myofilament energy (ATP) consumption, which coincided with improved cardiac contractility as demonstrated by improved dP/d*t*_max_ and dP/d*t*_min_ (Figure 4M), SBP, and left ventricular functional parameters, shown by pressure-volume loop hemodynamics (Supplemental Figure 3, C–G). Moreover, MI+Rest hearts showed improved intrinsic cardiac contractility independent of hemodynamic preload, as evidenced by the greater slope of the end-systolic pressure-volume relationship (Supplemental Figure 3G). Mechanistically, we observed a significant decrease in the phosphorylation of myosin binding protein C (MyBP-C), TnT, and tropomyosin (Tm) in MI+Rest hearts (Figure 4N), consistent with the reduced myofilament ATPase activity. Thus, rest helps to preserve cardiac contractile function after MI, which is important for protecting against progression to heart failure. Importantly, these data also revealed that rest benefited the heart in a disease phenotype–specific manner, remarkable precision medicine tailored to benefit different cardiac pathologies.

### Benefits of rest are independent of a functional circadian clock mechanism.

**Figure 5:**
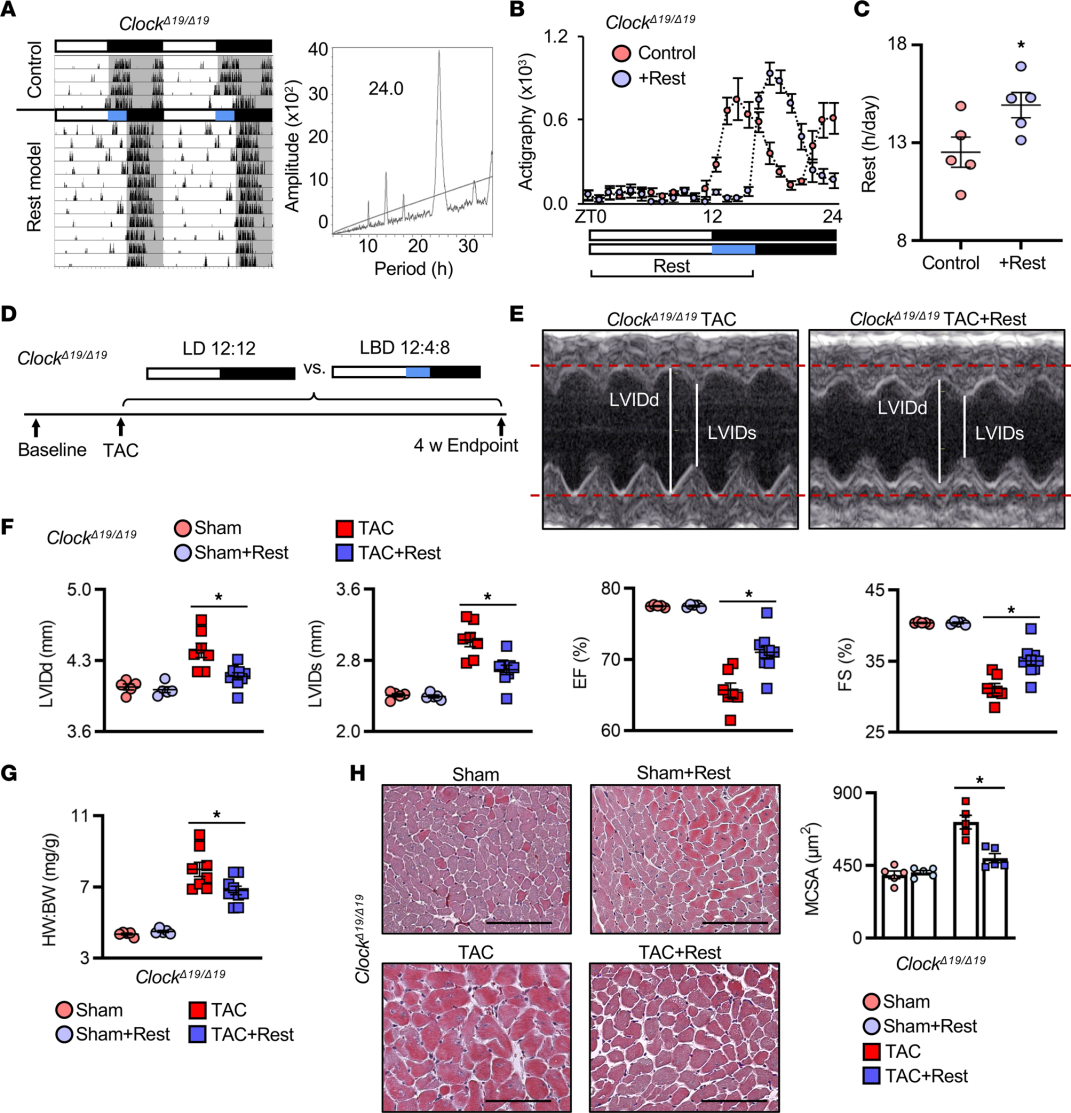
Benefits of rest are not dependent on a functional circadian mechanism. (**A**) Representative actigraphy and periodogram, (**B**) activity quantification, and (**C**) hours of rest per day in *Clock^Δ19/Δ19^* mutant mice under the rest model (+Rest) versus control. *n* = 5 mice/group. *P < 0.01, paired Student’s *t* test. (**D**) Rest model and cardiac hypertrophy experimental design in *Clock^Δ19/Δ19^* mice: mice underwent baseline echocardiography followed by TAC and were randomized to control conditions versus the rest model for up to 4 weeks. (**E**) Representative M-mode echocardiography showing that the cardiac benefits of rest persisted in *Clock^Δ19/Δ19^* mice, with (**F**) smaller LVIDd and LVIDs and better % EF and % FS and (**G**) smaller HW/BW ratio compared with controls at 4 weeks after TAC. *n* = 5 sham mice/group and *n* = 9 TAC mice/group. *P < 0.05, unpaired Student’s *t* test. (**H**) Representative images and quantification of cardiomyocyte cross-sectional area at 4 weeks after TAC. *n* = 5 hearts/group. *P < 0.005, unpaired Student’s *t* test. Scale bar: 100 μm.

In order to determine whether a functional circadian clock mechanism is necessary for rest-induced benefits to cardiac repair, we used *Clock*^Δ*19/Δ*19^ mutant mice, which importantly have intact behavioral responses to light yet disrupted central and peripheral clock gene cycling (35, 36). We found that the rest model delayed activity onset by 4 hours, while maintaining a normal approximately 24-hour period, as expected (Figure 5, A–C). Moreover, the *Clock*^Δ*19/Δ*19^ mice subjected to TAC (Figure 5D) benefited from the extended rest period, as demonstrated by echocardiography (Figure 5, E and F, and Supplemental Table 3), with less cardiac hypertrophy and smaller hearts (Figure 5G) and less cardiomyocyte hypertrophy (Figure 5H) compared with TAC control mice. These data suggest that the benefits of a brief period of extended morning rest are not functionally dependent on an intact clock mechanism, but rather likely driven by physiological outputs, such as autonomic modulation of blood pressure.

### Hemodynamic benefits of rest are mediated through sympathetic mechanisms.

**Figure 6:**
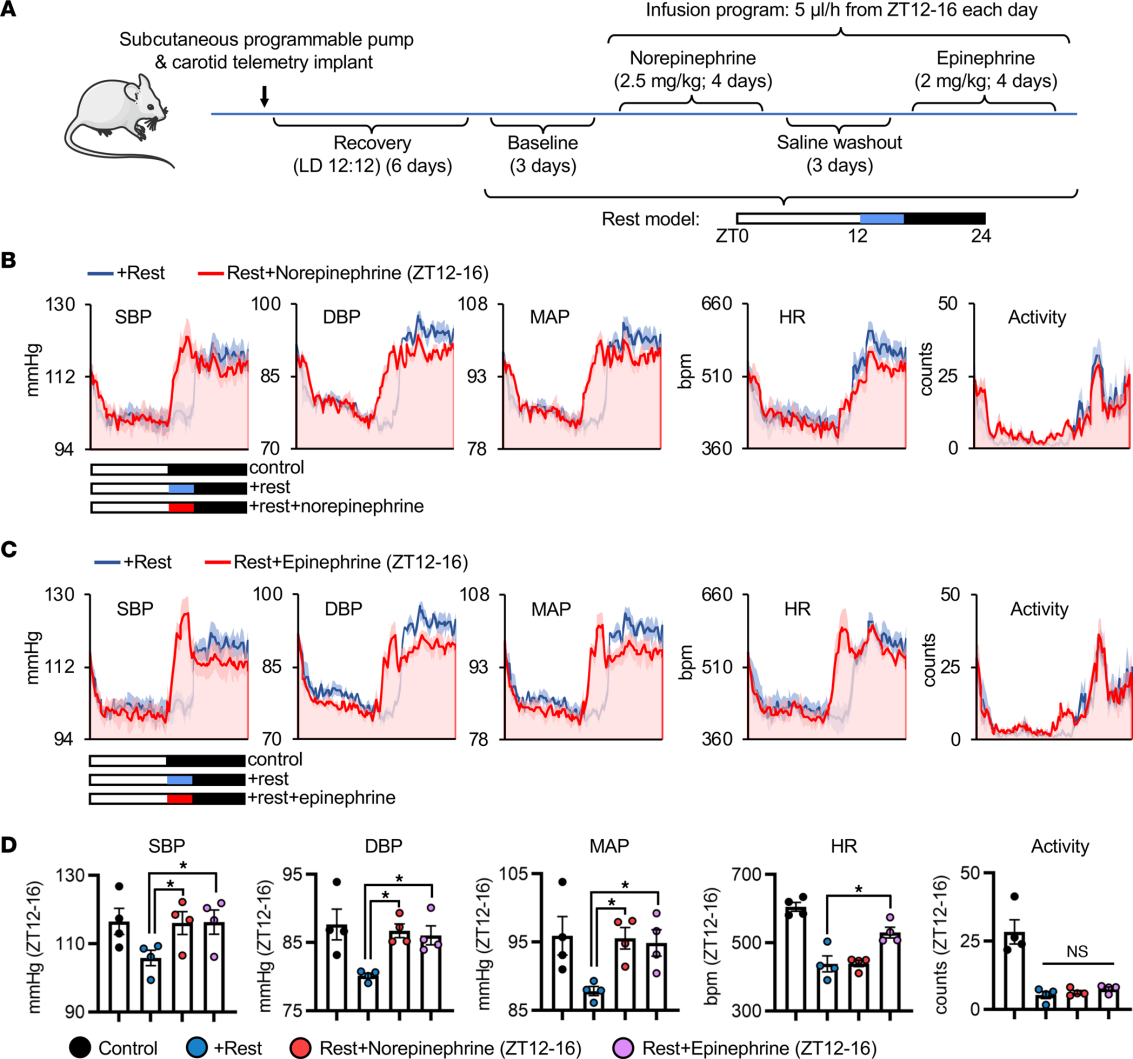
A brief period of morning rest delays the onset of sympathetic activity to benefit cardiovascular hemodynamics. (**A**) Schematic of experimental design. Healthy mice were implanted with both a subcutaneous programmable iPRECIO infusion pump and carotid artery radiotelemetry. After recovery in control LD 12:12, mice were placed under the rest model for baseline telemetry recordings, followed by timed infusion for ZT12–16 with sympathetic agonists norepinephrine and epinephrine. (**B**) Norepinephrine abates the hemodynamic benefits of rest, and similarly (**C**) epinephrine with (**D**) quantification across ZT12–16. *n* = 4 mice/group. *P < 0.05 by 1-way repeated-measures ANOVA and Tukey’s post hoc test.

Thus, we next examined the effects of rest on cardiovascular hemodynamics. We first performed loss-of-function experiments using timed dosing of sympathetic agonists and followed outcomes by *in vivo* radiotelemetry (Figure 6A). We used the iPRECIO programmable infusion pump system in order to time drug administration specifically over the 4-hour period of additional rest and implantable radiotelemetry to follow hemodynamics, with both approaches eliminating the stress of animal handling. We found that compared with baseline recordings under the rest model, mice that were then treated with the α-adrenergic receptor agonist norepinephrine over the 4-hour rest period showed a complete reversal of the effects of rest on SBP, DBP, and MAP, with no major change to heart rate or activity, as anticipated (Figure 6B). In a separate experiment, we treated the rest mice with the α- and β-adrenergic receptor agonist epinephrine and again demonstrated that sympathetic modulation reverses the effect of rest on SBP, DBP, and MAP (Figure 6C). Quantitative analyses over the rest period further confirmed these data (Figure 6D). Thus, the benefits of rest on cardiovascular hemodynamics appear to be driven by delaying early morning sympathetic activity.

### Rest-responsive pathways in experimental and human cardiovascular disease.

**Figure 7:**
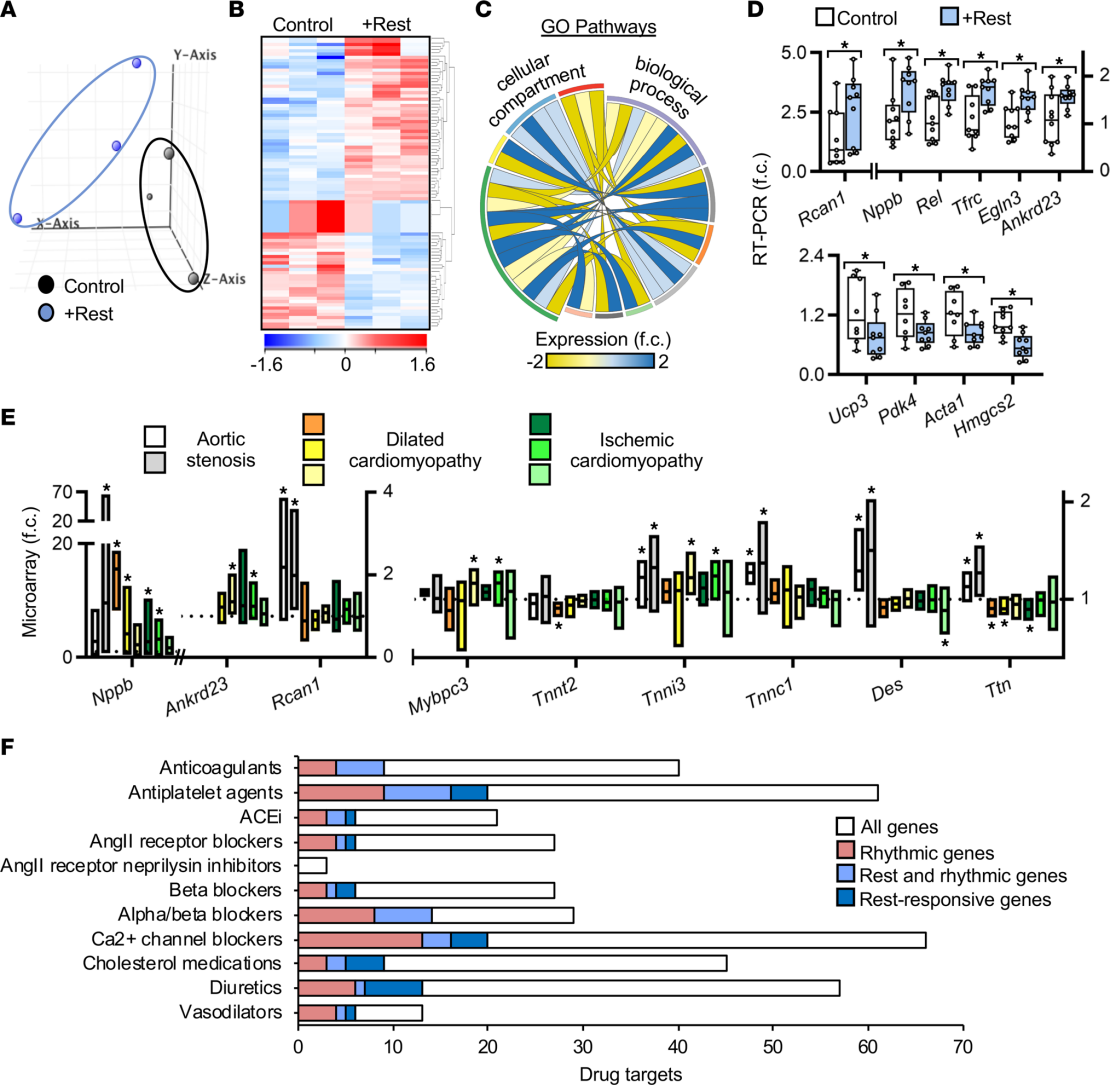
Rest regulates cardiac gene expression with implications for human heart disease. (**A**) Principal components analysis of cardiac transcriptome from healthy mice under the rest model (+Rest) for 4 weeks versus control. Hearts were collected during the middle of the rest period (ZT07). (**B**) Differentially regulated genes (fold change ≥ 1.3) in control versus rest model. (**C**) Gene ontology cellular compartment and biological process correlations, upregulated (blue) or downregulated (yellow) with rest. See Supplemental Figure 4. (**D**) Real-time PCR of cardiac biomarkers upregulated and downregulated in the heart with rest, pooled across the rest period (ZT03, 07, 11). *n* = 9/group from *n* = 3/time point, *P < 0.05, unpaired Student’s *t* test. (**E**) Open-source human heart tissue gene bioinformatics of cardiac remodeling and myofilament genes. *P < 0.05 versus nonfailing controls, unpaired Student’s *t* test. (**F**) Rhythmic and rest-responsive genes encode molecular drug targets of commonly prescribed cardiac medications.

Evidence thus far suggests that rest delayed early morning autonomic activity to benefit the remodeling heart. To elucidate a molecular basis for this, we first used mRNA arrays and quantified the cardiac transcriptomes of healthy mice under the rest model versus the control. Beginning with a background set of 28,137 known protein-coding mouse transcripts, principal components analysis clearly identified 2 groups of global gene expression between control and extended rest hearts (Figure 7A). From this, we defined “rest gene” as any gene in the extended rest group identified as robustly expressed on GeneSpring analyses with at least a greater than 1.3-fold change versus control hearts. In this context, we identified 91 rest genes in the heart (Figure 7B and Supplemental Table 4). Next, we used the Gene Ontology database as a basis for our pathway network and found many rest genes encode critical regulators of cardiac growth, renewal, and remodeling (Figure 7C and Supplemental Figure 4); for example, regulator of calcineurin 1 (*Rcan1*) (37), natriuretic peptide B (*Nppb*) (38), REL proto-oncogene, NF-κB subunit (*Rel*) (39), transferrin receptor (*Tfrc*) (40), Egl-9 family hypoxia inducible factor 3 (*Egln3*) (41), ankyrin repeat domain 23 (*Ankrd23*) (42), uncoupling protein 3 (*Ucp3*) (43), pyruvate dehydrogenase kinase 4 (*Pdk4*) (44), actin alpha 1 (*Acta1*) (45), and 3-hydroxy-3-methylglutaryl-CoA synthase 2 (*Hmgsc2*) (46), all of which play critical roles in the pathophysiology of cardiovascular diseases. These biologically significant rest genes were validated by real-time PCR (Figure 7D).

To test for human relevance, we turned to microarray data sets of control and failing human hearts (Supplemental Table 5). Strikingly, the rest genes and sarcomere gene products identified in our experimental rest studies are clear biomarker targets in human heart disease (Figure 7E), supporting the notion that rest can benefit both experimental and clinical cardiovascular disease conditions. Seizing upon the recent observation that many clinical drugs target rhythmic gene products (47), we observed that many of our identified rest genes also exhibited an early morning pattern (Figure 7F and Supplemental Table 6), which helps to explain how the heart benefits from early morning rest and provides new potential pharmacological targets for treatment of disease. We further investigated best-selling and commonly taken heart drugs targeting rest gene pathways. Notably, two-thirds of the top prescribed cardiovascular drugs listed by the American Heart Association target rest-responsive genes (Figure 7F and Supplemental Table 6), leading us to speculate that drug efficacy can be improved upon in conjunction with an additional brief period of daily rest.

## Discussion

**Figure 8:**
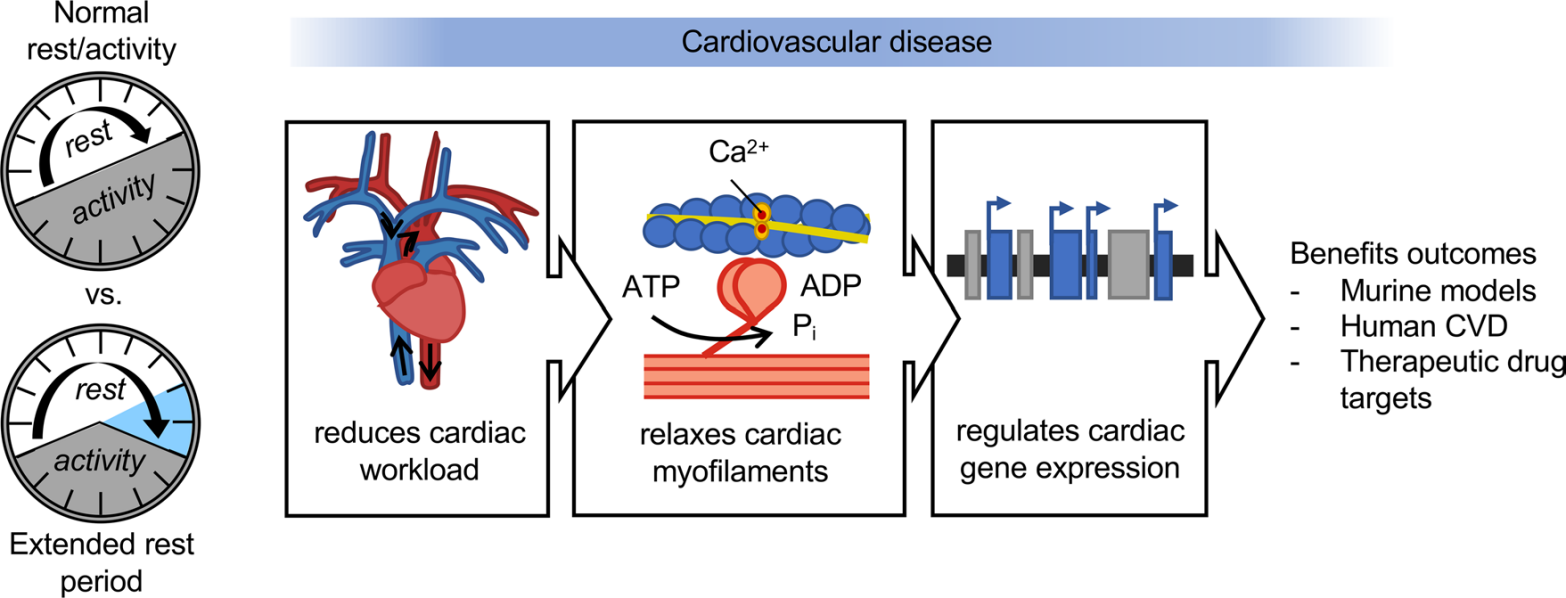
A mouse model of extended rest reveals how rest reduces cardiac hemodynamic workload, relaxes the cardiac myofilaments, and regulates cardiac gene profiles to benefit outcomes in cardiovascular disease.

The data presented here are consistent with the hypothesis that rest plays a key role in cardiac remodeling and improves outcomes. Given the limitations of current preclinical models to study rest, we developed a murine model that allows us to recapitulate the clinical phenotypes of human heart disease and study the physiological and molecular benefits of rest on cardiac repair. Here, we found that briefly extending the daily rest period played a critical role in triggering pro-cardiac responses. Using multiple approaches in preclinical models of heart disease and human gene data from patients with cardiovascular disease, we uncovered evidence implicating rest as benefiting cardiac structure and function. Rest-induced reduction of the onset of morning sympathetic activity lowered hemodynamic stress, preserved contractile function at the level of the cardiac sarcomere, and acted in a precision therapy manner such that it was disease phenotype specific. We also identified rest genes that have morning rhythmicity and underlie human heart disease and are common targets of drug therapies (Figure 8). To further elucidate the physiological mechanisms underlying the benefits of rest on cardiac remodeling, future investigations are warranted, such as examining long-term cardiac outcomes in mice with gain and loss of function of sympathetic outputs over the rest period. Ultimately, our findings implicate a brief period of morning rest as a master mediator of pathways critical for benefiting cardiac repair.

This work outlines a cassette of rest-responsive genes, which have important roles in cardiac growth and remodeling and may underlie the molecular mechanisms mediating the beneficial effects of rest on the heart. For example, *Rcan1* expression is driven by and reciprocally regulates calcineurin signaling in cardiac hypertrophy and shows remarkable time-of-day variation in expression (37). *Nppb* encodes a well-established biomarker of heart failure (BNP/NT-proBNP) but also plays important roles under normal physiological conditions in regulating blood pressure/volume, cell proliferation, and inflammation (38). *Rel* encodes the c-Rel subunit of NF-κB, which plays a critical role in cardiac remodeling and hypertrophy (39). *Tfrc* has been shown as an essential cardiac iron uptake receptor, with loss of expression leading to death by 2 weeks of age in mice (40), suggesting a critical role in healthy cardiac physiology. *Egln3*, also known as PHD3, is responsible for regulation of HIF-1α signaling in the heart and may play a role in regulating cardiomyocyte apoptosis under hypoxia (41). *Ankrd23* (DARP), is part of a family of mechanosensitive proteins in the sarcomere, which translocate to the nucleus during cell stretch to regulate stretch-dependent gene expression (42). Interestingly, several metabolism related genes were observed to be downregulated in the resting heart, including *Ucp3*, *Pdk4*, and *Hmgcs2*, which play important roles in regulating mitochondrial energy metabolism and oxidative stress (43), regulating fatty acid versus glucose oxidation in the heart (44), and regulating cardiac ketogenesis (46), respectively. Moreover, we observed downregulation of *Acta1*, well-established to be upregulated in hypertrophic cardiomyopathies (45), in the resting heart. Collectively, rest induces diverse changes in the cardiac milieu, which may underlie the key molecular mediators of the benefits of rest in cardiovascular disease.

In these studies, we used experimental murine models of cardiovascular disease to assess the beneficial effects of rest on the cardiac repair. Unlike in humans, the mice used in these experiments were young and otherwise healthy with no comorbidities. Nevertheless, the day-night variation in hemodynamic and sympathetic nervous system activity (although of opposite diurnal phase) is consistent with that found in humans and large animal models. There is a clear rise in both sympathetic drive and hemodynamic parameters at the start of the wake period in both rodents (48) and humans (28). Regardless, key physiological (sympathetic/hemodynamic) and molecular responses applicable to a wide range of disease conditions in humans also exhibit a profound day-night variation, and may be mitigated by early morning rest similar to that demonstrated here.

Strategies to add a brief daily period of rest can be applied alongside and to elevate contemporary therapies for heart disease. Our observations are certainly not conclusive for human patients, but they have enormous implications for benefitting treatment of cardiovascular disease, a leading cause of death worldwide. A further consideration is how to promote rest in humans; in the murine models we used light, but in humans we would require alternative strategies. Based on our findings, it is tempting to speculate that nighttime dosing of long-acting or delayed-release sympathetic antagonist therapies paired with an extra hour in bed each morning may collectively delay the early morning rise in sympathetic activity and hemodynamic stress and limit adverse cardiac remodeling. While it is beyond the scope of the study, future investigations using preclinical diurnal models (e.g., porcine; ref. 49), which can recapitulate human cardiovascular physiology, would be interesting to assess these approaches and efficacy in a diurnal model. We hope that our preclinical study will stimulate the initiation of clinical trials reappraising the value of rest as an important long-term lifestyle approach, leading to longer and healthier lives.

Using a remarkably simple strategy, we developed an evidence-based murine model to study the benefits of promoting brief daily rest as cardiovascular disease therapy, especially during the critical early period of cardiovascular disease pathogenesis and cardiac repair, which prompts further investigation into potential applications to healing from many clinical diseases.

## Methods

### Experimental animals.

All studies were carried out in accordance with the guidelines of the Canadian Council on Animal Care and were reviewed and approved by the University of Guelph IACUC. All animals were housed at the University of Guelph Central Animal Facility. Standard rodent chow and water were provided *ad libitum*. Rest and activity were recorded from 8-week-old male WT C57BL/6N mice (Charles River Laboratories) or *Clock*^Δ*19/Δ*19^ mice homozygous for the CLOCK point mutation (35) and bred on a C57Bl/6 background. Mice were maintained at the University of Guelph Central Animal Facility using individual cages equipped with running wheels (Colbourn Instruments) as described (21, 36), and data were collected and analyzed using ClockLab Software (Actimetrics). Rest was measured as hours per day with less than 100 counts of activity. Animals were placed on (a) a control (normal LD 12:12) or (b) an extended light cycle to induce rest using white light (LD 16:8) or (c) an extended period of light under red light (>620 nm wavelength; LR 12:12) or (d) “extended rest” induced by blue light (420–520 nm wavelength, LBD 12:4:8, rest model) or (e) constant darkness (DD). Light wavelengths were filtered using blue Roscolux 74 or red Roscolux 27 (Rosco Laboratories) over white fluorescent lights (Octron T8; Sylvania). Cardiac hypertrophy was surgically induced by TAC as previously described (16–18, 36). MI was surgically induced by left anterior descending coronary artery ligation as described (19, 50). All surgeries were performed between zeitgeber time (ZT) 01 and ZT04 to avoid confounding circadian effects and used the same methods for anesthesia, intubation, and analgesia as previously described, and mice were given a subcutaneous injection of buprenorphine (0.1 mg/kg) for postoperative analgesia upon awakening and at 8 hours and 24 hours postoperatively. After recovery, starting on the first night after surgery, mice were housed under control or extended light regimens for up to 8 weeks.

### Echocardiography.

Cardiac structure and function were assessed at baseline and 1 and 4 weeks after TAC or at baseline and 1, 4, and 8 weeks after MI using a GE Vivid e90 ultrasound machine (GE Medical Systems) with an L8-18i-D 15 MHz linear array transducer under light anesthesia (1.0% isoflurane). Images were analyzed on an offline system using EchoPAC (GE Medical Systems). Measurements were taken at the midpapillary level and used to determine LVIDd, LVIDs, % EF, % FS, interventricular septal wall thickness at diastole, left ventricular posterior wall thickness at diastole, and heart rate. For the WT TAC studies, a total of 44 mice were used (*n* = 11 mice/group). For the MI studies, 20 mice were used (*n* = 10 mice/group). For the *Clock*^Δ*19/Δ*19^ TAC studies, a total of 26 mice were used (*n* = 5 mice/sham group; *n* = 7–9 mice/TAC group). At least 5 images per animal were used for analysis, and means are presented.

### In vivo pressure-volume hemodynamics.

At 8 weeks after MI, mice were anesthetized with 4% isoflurane, intubated, and ventilated. A 1.2Fr pressure-volume catheter (Transonic) was inserted via the right carotid artery and advanced into the ascending aorta for blood pressure measurements. The catheter was then advanced into the left ventricle (LV). Real time physiological LV and aortic pressure measurements were recorded on an ADInstrument PowerLab, including LV end-systolic pressure, LV end-diastolic pressure, LV end-systolic volume, LV end-diastolic volume, stroke volume, cardiac output, maximum and minimum first derivatives of LV pressure (dP/d*t*_max_; dP/d*t*_min_), SBP, and DBP. MAP was calculated as DBP + (SBP–DBP)/3. After PV measurements were obtained, the inferior vena cava was briefly occluded to block venous return to determine the end-systolic PV relationship (51). Continuously recorded pressures were analyzed with Lab Chart 7 (Colorado Creeks). A total of 18 mice were used (*n* = 9 mice/group), with data reported as mean ± SEM.

### In vivo radiotelemetry.

Diurnal cardiovascular hemodynamics were assessed using PA-C10 murine telemetry probes (Data Sciences International) to collect continuous blood pressure and heart rate data from conscious, freely moving mice, as previously described (17, 36). Animals were anesthetized with 4% isoflurane, intubated, ventilated (model 687; Harvard Apparatus), and maintained at 2.5% isoflurane throughout the procedure. The right carotid artery was isolated, and the telemeter catheter was implanted to the level of the aortic arch via the carotid artery. The telemeter transmitter unit was implanted in a subcutaneous skin pouch, and the neck incision was closed using silk 6-0 suture (Covidien). Mice were administered buprenorphine (0.1 mg per kg) analgesia upon awakening and at 8 hours and 24 hours postoperatively. Recordings were initiated at 1 week after telemeter implantation, and measurements were collected over 3 continuous 24-hour cycles for each light condition. After baseline telemetry recordings, mice were subjected to TAC surgery. At 4 weeks after TAC, measurements were collected over 3 additional days under each light cycle. SBP, DBP, MAP, heart rate, and activity were analyzed using the Data Quest IV system (Data Sciences International). Measurements were taken every 5 minutes for 30 seconds and averaged into 1-hour bins according to ZT. A total of 4 mice were used, enabling a paired analysis of the same mice before and after all experimental interventions, with data reported as mean ± SEM.

### Histology.

At 4 weeks after TAC or 8 weeks after MI following hemodynamic assessments, mice were euthanized with isoflurane and cervical dislocation. BW, HW, and tibia length were measured for each animal. Hearts were removed, perfused with 1 M KCl, fixed in 10% neutral buffered formalin for 48 hours, and paraffin embedded. Hearts were then sectioned at 5 μm thickness from apex to base, collecting 10 sections every 300 μm. Sections were stained with Masson’s trichrome, mounted using Cytoseal 60 mounting media (Thermo Fisher Scientific), visualized with a Nikon E600 microscope using Q-Capture software (QImaging), and quantified using ImageJ (NIH). Myocyte cross-sectional area was quantified from 100 or more cardiomyocytes/heart at the midpapillary level over at least 5 separate fields of view (52). Infarct thickness was determined from a minimum of 5 measurements/section over equidistant points along the infarct region. LV diameter was determined from sections collected at midpapillary level. Relative infarct size was determined by dividing the sum of the endocardial and epicardial circumference occupied by the infarct by the sum of the total LV epicardial and endocardial circumferences (21, 50). For the WT TAC studies, a total of 15 mice were used (*n* = 3 mice/sham group; *n* = 4–5 mice/TAC group). For the MI studies, 10 mice were used (*n* = 5 mice/group). For the *Clock*^Δ*19/Δ*19^ TAC studies, a total of 20 mice were used (*n* = 5 mice/group). Data are presented as mean ± SEM.

### Myofilament isolation, actomyosin MgATPase assay, and protein phosphorylation.

At 4 weeks after surgery, TAC, MI, and sham animals were euthanized by isoflurane and cervical dislocation at ZT15. Hearts were collected, snap-frozen in liquid nitrogen, and stored at –80°C until use. Cardiac myofilaments were isolated by differential centrifugation using the protocol described by Podobed et al. (34). Actomyosin MgATPase activity in isolated cardiac myofilaments was determined using a modified Carter assay as described previously (19, 34). Isolated myofilament proteins were separated using 12% SDS-PAGE, and protein phosphorylation levels were quantified using the PRO-Q Diamond phosphoprotein gel stain (Invitrogen) by following the protocol of Podobed et al. (34). Gels were then stained with Coomassie to determine total protein. Gel imaging was performed on a Bio-Rad ChemiDoc MP Imaging System (Bio-Rad) and analyzed using ImageJ (NIH) with protein phosphorylation normalized to total protein. Samples were run, stained, imaged at the same time on separate gels, and normalized to actin; see complete unedited blots in the supplemental material.

### Programmable infusion pump implantation and sympathetic agonist administration.

Eight-week-old male mice (~25 g) were anesthetized with 4% isoflurane, intubated, ventilated, and maintained at 2.5% isoflurane throughout the procedure. Telemetry device implantation into the carotid artery was performed as described above. Programmable iPRECIO pumps (Primetech Corp.) (53) were implanted into a subcutaneous skin pouch on the dorsal side of the mouse with a 17 mm catheter fixed with a 6-0 suture to the subcutaneous muscle layer. Animals were recovered and postoperative care performed as described above. Mice were maintained for 6 days under control LD 12:12 conditions to allow for normal diurnal rhythms in hemodynamic parameters to recover, followed by 3 days under the rest model (LBD 12:4:8) to collect baseline hemodynamic measurements. During recovery and baseline recordings, pumps were programmed to infuse saline at a constant rate of 0.1 μL/h to maintain pump function and catheter flow. Pumps were then refilled with the α-adrenergic receptor agonist norepinephrine at a dose of 2.5 mg/kg/day (54) and programmed to infuse 5 μL/h for ZT12–16 for 4 days while mice were maintained under the rest model. Pumps were then rinsed out with saline and refilled with saline solution for a washout period of 3 days under the same infusion settings. After the washout period, pumps were refilled with the α- and β-adrenergic receptor agonist epinephrine at a dose of 2 mg/kg/day (55), while still programmed to infuse 5 μL/h for ZT12–16 for 4 additional days under the rest model. SBP, DBP, MAP, heart rate, and activity were continuously recorded throughout the infusion experiment using the Data Quest IV system (Data Sciences International). Measurements were taken every 5 minutes for 30 seconds and averaged into 15-minute bins according to ZT. A total of 4 mice were used, enabling a paired analysis of the same mice before and after all experimental interventions, with data reported as mean ± SEM.

### RNA isolation, microarray, and bioinformatics analyses.

After 4 weeks under either the rest model or control conditions, healthy mice were euthanized by isoflurane and cervical dislocation at 4-hour intervals over 24 hours (ZT03, 07, 11, 15, 19, and 23). Hearts were collected, snap-frozen in liquid nitrogen, and stored at –80°C until use. Total RNA from murine heart tissue was isolated using the miRNeasy Mini Kit (Qiagen), as previously described (36). RNA quantity and quality were assessed by Nanodrop (260/280 ≥ 2; Thermo Fisher Scientific) and RNA ScreenTape (RIN ≥ 7; Agilent). Whole-genome microarray analyses were performed using the Affymetrix GeneChip Mouse Gene 2.0 ST array, which interrogates 35,240 RefSeq coding and noncoding transcripts (Gene Expression Omnibus [GEO], accession GSE115567). Gene expression analyses were performed on 6 individual mouse heart samples. Bioinformatics analyses were performed using GeneSpring GX v14.9 (Agilent Technologies). Raw.cel files were loaded into a project file with exon analysis and Affymetrix exon expression experiment settings and a biological significance workflow analysis. The most recent mouse gene 2.0 ST array annotation technology (MoGene-2_0-st_na36_mm10_2016-07-06) was used to perform all analyses. Raw fluorescence data were normalized across all chips using the exon robust multiarray summarization algorithm. Experiment parameters for sample groups and replicate structure were defined and launched as a group-level interpretation. Quality control across all samples was assessed by log_2_(normalized signal values) expression of 8 control hybridization probes across all chips, and group-level clustering was analyzed by principal components analysis. The probe-set filter parameter was defined to include all 34,351 probe-set entities, then filtered by expression using a lower cutoff of 60 raw fluorescence units. Differentially expressed genes were determined by fold-change analysis of all entities with 1.3 or greater fold change in expression between rest versus control hearts (Supplemental Table 4). This is consistent with our previous studies, which showed that this microarray analysis cutoff can be further validated by subsequent real-time PCR methods (20, 52). Hierarchical cluster analysis of this gene cassette generated heatmaps of entity expression relative to control hearts. Gene Ontology analysis was performed on differentially expressed gene lists using the Database for Annotation, Visualization, and Integrated Discovery (DAVID) Functional Annotation Tool (DAVID Bioinformatics 6.8, NIAID/NIH) (56). Circos plots were generated using Circos v.0.69-9 (57).

### Quantitative real-time PCR.

Analysis of mRNA expression by quantitative PCR (qPCR) was performed on a ViiA7 real-time PCR system (Applied Biosystems) using the Power SYBR Green RNA-to-Ct one step kit (Life Technologies) under the following protocol: reverse transcription at 48°C for 30 minutes and 95°C for 10 minutes for 1 cycle, followed by amplification at 95°C for 15 seconds and 60°C for 1 minute for 40 cycles, followed by hold at room temperature. The following real-time PCR primer sequences were used, including forward and reverse sequences for each gene, respectively: *Rcan1* exon 4 isoform mouse (5′–3′) CCCGTGAAAAAGCAGAATGC, TCCTTGTCATATGTTCTGAAGAGGG; *Nppb* mouse GCGAGACAAGGGAGAACAC, GCGGTGACAGATAAAGGAAAAG; *Rel* mouse CAGAGTGACTTCAAGGGAAC, GTTAGGCACCGAGTCTTTAG; *Tfrc* mouse CACTCGCCCAAGTTATATCC, GCACGGTGATACTCATACTG; *Egln3* mouse CCCGAACTCTGTACGAAAC, CTGCTTGTGGGATTCTAGC; *Ankrd23* mouse TCCAGGGCATGAGAGAAG, GGCTGCTACTGGTAGAAATG; *Ucp3* mouse CCCAACATCACAAGAAATGCC, ACAGAAGCCAGCTCCAAAG; *Pdk4* mouse CGCCAGAATTAAACCTCACAC, TTCTTGATGCTCGACCGTG; *Acta1* mouse AGACCTTCAACGTGCCTG, CGTCCCCAGAATCCAACAC; *Hmgcs2* mouse CACCTGCTACTAACCTTG, CAAGAGGACACTTTCAGG; *Bmal1* mouse CCAGGGTTTGAAGTTAGAGTCC, TGAAGTCGCTGATGGTTGAG; *Clock* mouse GCCTCAGCAGCAACAGCAGC, ACCGCATGCCAACTGAGCGA; *Per2* mouse TCATCATTGGGAGGCACAAA, GCATCAGTAGCCGGTGGATT; *Cry1* mouse ACAGAGGGCTAGGTCTTC, GCTACAACTCGGGACATTC; *Nr1d1* mouse GGGCACAAGCAACATTACCA, CACGTCCCCACACACCTTAC; *Dbp* mouse GAACCCATCCATCCAACC, CCAGTAGCTGCTTTGTCC; and histone mouse GCAAGAGTGCGCCCTCTACTG, GGCCTCACTTGCCTCCTGCAA. Relative gene expression was normalized to histone using the ΔΔCt method as described previously (19, 20).

### Human myocardial gene expression microarray analyses.

Human myocardial gene expression data were obtained using publicly available data sets from the GEO database (58). Raw.cel microarray files were downloaded from 6 independent data sets examining myocardial gene expression from LV biopsies from a total of 140 patients. Data sets included patients with aortic stenosis (AS) (59), dilated cardiomyopathy (DCM) (60–62), ischemic cardiomyopathy (ICM) (61–63), and nonfailing controls (GEO data sets: GSE1145 [AS], GSE10161 [AS], GSE3585 [DCM], GSE42955 [DCM, ICM], GSE79962 [DCM, ICM], GSE16499 [ICM]; see Supplemental Table 5). Raw microarray files were analyzed from each data set using GeneSpring GX v14.9 (Agilent Technologies Inc.) as described above. All probe-set entity lists were then interrogated for rest-responsive genes identified from our murine studies and analyzed as fold-change in expression from nonfailing control hearts. See Supplemental Table 5 for *n* values and microarray technologies for each data set.

### Cardiac medications targeting rest pathways.

A list of the top 66 commonly prescribed cardiovascular medications was obtained from the American Heart Association website (64). All molecular drug targets were determined using the DrugBank database (65). Lists were then curated for target genes with known rhythmic transcripts (JTK_CYCLE *P* < 0.05) based on analyses from mouse heart tissue using the CircaDB database (66) and for rest-responsive genes identified from our rest model microarray analyses in the murine heart.

### Statistics.

Values are presented as mean ± SEM. Statistical comparisons were made by paired or unpaired 2-tailed Student’s *t* test or by 1-way repeated-measures ANOVA and Tukey’s post hoc test, as applicable. All analyses were performed using GraphPad Prism 8 or Microsoft Excel. A *P* value of 0.05 or less was considered statistically significant. All endpoints, *n* values, and statistics are provided in detail in the figure legends and Supplemental Material.

### Study approval.

All studies were carried out in accordance with the guidelines of the Canadian Council on Animal Care and were reviewed and approved by the University of Guelph IACUC.

## Author contributions

CJR and TAM conceptualized the study and designed the experiments. CJR, MR, FJA, TNK, WGP, and TAM performed experiments and analyzed and/or interpreted the experimental results. CJR and TAM prepared the figures and drafted the manuscript. All authors have read and given permission to the paper.

## Supplementary Material

Supplemental data

## Version 1 10/18/2022

In-Press Preview

## Version 2 11/22/2022

Electronic publication
