# Supplementary material for: A brief morning rest period benefits cardiac repair in pressure overload hypertrophy and postmyocardial infarction
Source: JCI Insight. 2022 Nov 22;7(22):e164700. doi: 10.1172/jci.insight.164700 (PMC9746813; doi:10.1172/jci.insight.164700)
Supplement: Supplemental data [file jciinsight-7-164700-s256.pdf]

Supplemental Material

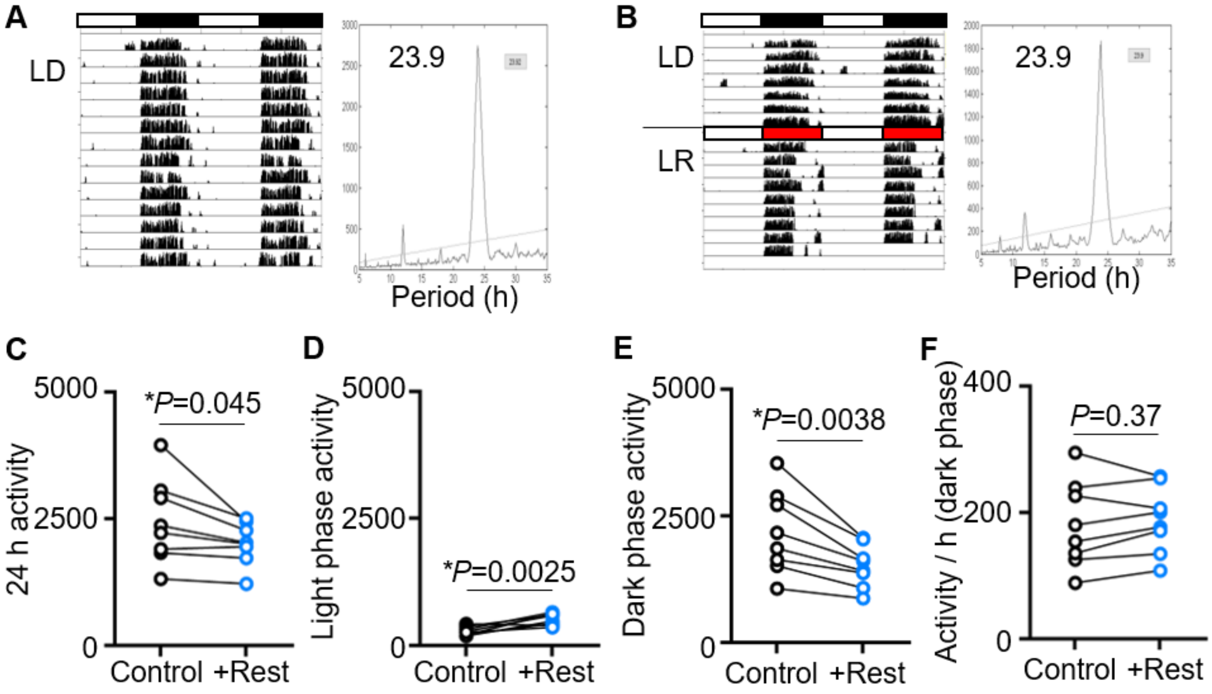

Supplemental Figure 1. Day-night activity rhythms. (**A**) Running wheel actigraphy (left) and periodogram quantification (right) of 24 h locomotor activity under a normal LD 12:12 cycle, and (**B**) similar activity profiles under 12 h light: 12 h red light (>620 nm) (LR). n=5 mice/group. (**C**) Radiotelemetry data of total 24 h activity, (**D**) light phase activity, (**E**) dark phase activity, and (**F**) activity/hour over the dark phase, in healthy mice under control LD 12:12 or the rest model. n=8 mice/group. *$P<0.05$ by paired Student's *t*-test.

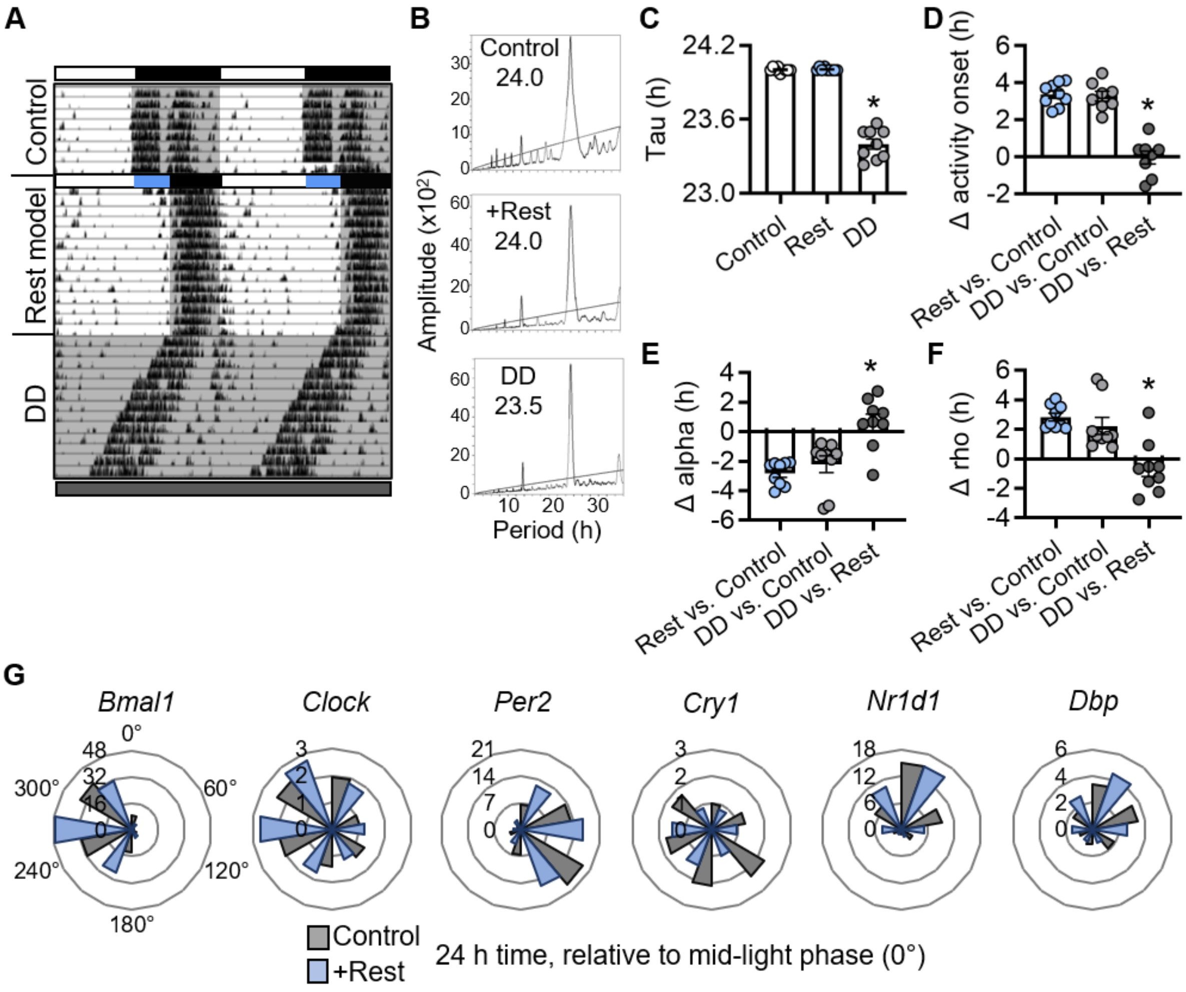

Supplemental Figure 2. Physiologic and molecular rest patterns. (**A**) Double-plotted actogram showing representative running wheel recording of mice under control (LD 12:12), the rest model (+Rest; LBD 12:4:8), and release into constant darkness (DD). Shaded areas represent the dark period. (**B**) Representative periodograms under control conditions (top), rest model (middle), and DD (bottom). (**C**) Quantitative analysis of tau, (**D**) change in activity onset, (**E**) change in alpha (active period), and (**F**) change in rho (inactive period), between control, +Rest, and the first 5 days of DD. n=9 mice, **P*<0.005 vs. all other groups, by one-way repeated measures ANOVA and Tukey post-hoc test. (**G**) Radar plots of cardiac mRNA expression of core circadian clock mechanism genes in mice housed under control or the rest model (+Rest). Expression data (measured as qRT-PCR fold change) are plotted relative to the middle of the light phase under either control or rest conditions. n=3 mice/timepoint/group. Data are represented as mean ± s.e.m.

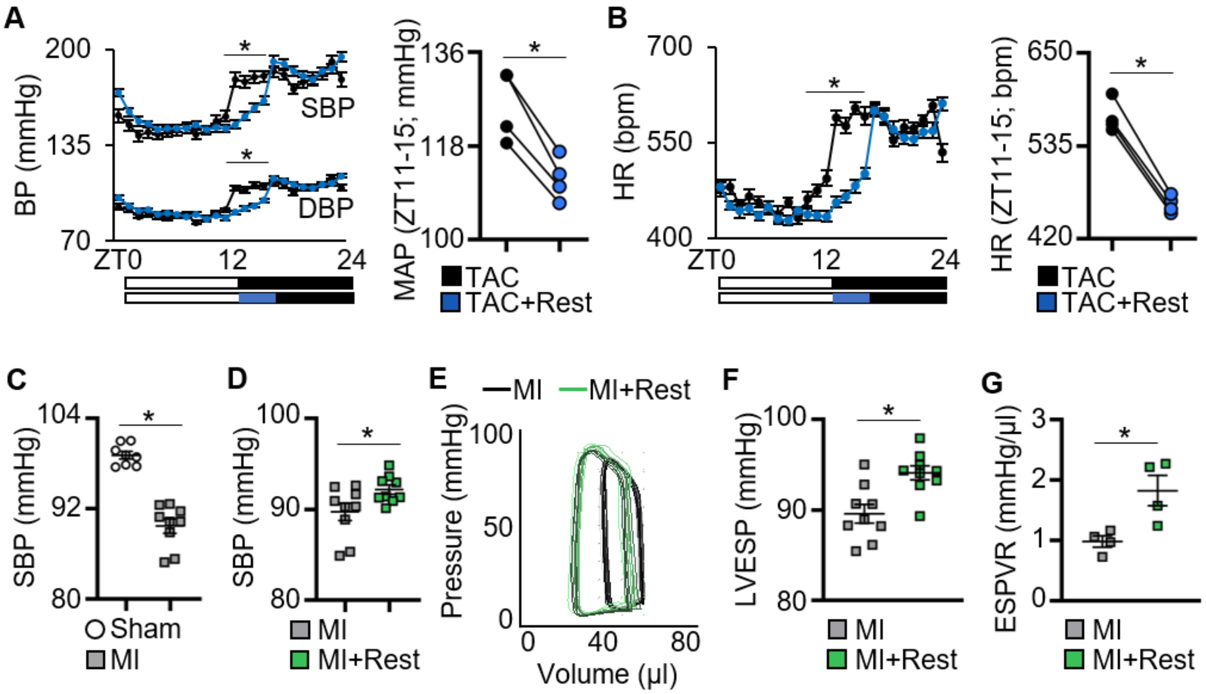

Supplemental Figure 3. Rest alters cardiovascular hemodynamics to benefit cardiac contractility in a disease phenotype-specific manner. (**A**) A short period of extended rest lowers cardiac afterload, as evidenced by diurnal systolic (SBP) and diastolic (DBP) blood pressure and quantification of MAP and (**B**) lowers cardiac workload, shown by reduced heart rate (HR) during rest, in TAC+Rest hearts vs. TAC controls, at 4 weeks post-surgery. n=4 mice/group, *P<0.005 TAC vs. TAC+Rest, paired Student's *t*-test. (**C**) MI lowers SBP as compared to sham mice, and (**D**) this is restored in mice maintained under the rest model up to 8 weeks post-MI. (**E**) MI+Rest hearts show better pressure-volume hemodynamic profiles in vivo, including (**F**) greater left ventricular end systolic pressures (LVESP), and (**G**) this is independent of preload, as evidenced by greater slope of the end-systolic pressure volume relationship. n=9 mice/group. *P<0.05 MI vs. MI+Rest, by unpaired Student's *t*-test. Data are represented as mean ± s.e.m.

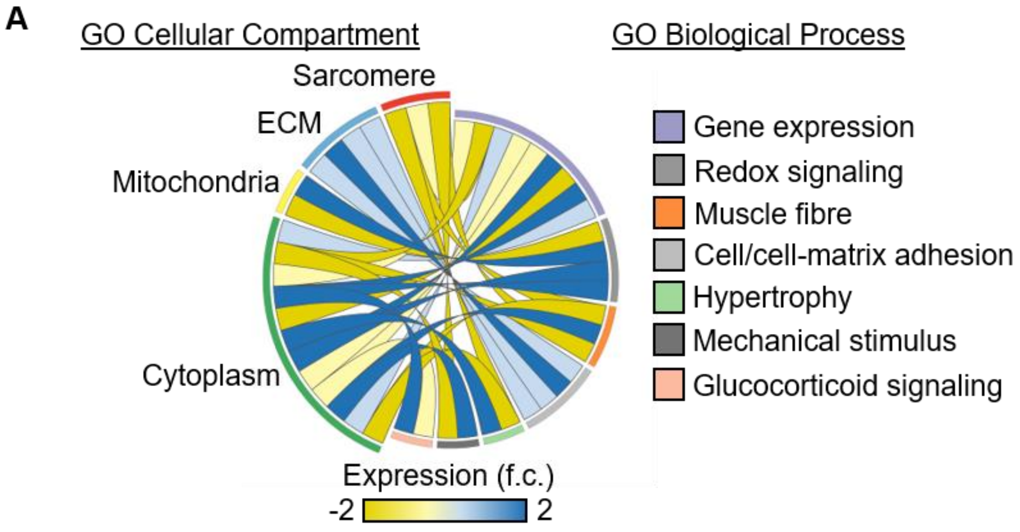

Supplemental Figure 4. Rest gene biomarkers in the heart. (**A**) Gene ontology (GO) cellular compartment and biological process correlations, illustrates genes up-regulated (blue) or down-regulated (yellow) in the heart with rest. Circos plot visualizes key biological processes regulated by rest that are involved in cardiac growth, repair, and remodeling and links to the subcellular compartments where these pathways are localized. ECM = extracellular matrix. n=3 hearts/group for microarray transcriptomics and bioinformatics analyses.

Supplemental Table 1. Rest limits adverse cardiac remodeling in pressure overload-induced cardiac hypertrophy in mice.

	TAC+Rest	TAC	Sham+Rest	Sham
**Echocardiography (Baseline)**				
LVIDd (mm)	4.01±0.02	4.02±0.02	4.01±0.03	4.00±0.02
LVIDs (mm)	2.41±0.02	2.43±0.04	2.42±0.02	2.39±0.04
EF (%)	76.89±0.39	76.31±0.75	76.42±0.22	77.03±0.85
FS (%)	39.93±0.34	39.48±0.69	39.58±0.16	40.17±0.77
HR (bpm)	480±9	443±7	470±14	457±15
**Echocardiography (4 weeks)**				
LVIDd (mm)	4.28±0.07**	4.97±0.15	4.03±0.03	4.04±0.03
LVIDs (mm)	2.80±0.11**	3.74±0.18	2.40±0.03	2.42±0.02
EF (%)	70.25±1.87**	55.55±2.40	77.37±0.45	77.04±0.30
FS (%)	34.75±1.32**	25.13±1.40	40.34±0.40	40.04±0.26
IVSd (mm)	0.67 0.01*	0.70 0.01	0.62±0.01	0.62±0.01
LVPWd (mm)	0.67 0.01*	0.71 0.00	0.61±0.01	0.62±0.00
HR (bpm)	465±8	469±9	484±8	476±9
**Morphometry (4 weeks)**				
HW (mg)	142.36±6.61**	193.55±8.47	115.45±4.33	114.64±4.01
HW:BW (mg/g)	5.67±0.23**	7.16±0.27	4.13±0.04	4.09±0.09
HW:TL (mg/mm)	7.34±0.39**	9.82±0.44	5.84±0.23	5.77±0.20

LVIDd, left ventricle internal dimensions at diastole; LVIDs, LV internal dimensions at systole; % EF, % ejection fraction; % FS, % fractional shortening; HR, heart rate; IVSd, interventricular septal wall thickness at diastole; LVPWd, left ventricular posterior wall thickness at diastole; HW, heart weight; HW:BW, HW:body weight ratio; HW:TL, HW:tibia length ratio. *P<0.05, **P<0.001 TAC control vs. TAC+Rest, by unpaired Student's *t*-test. n=11 mice/group. Data are mean ± s.e.m.

Supplemental Table 2. Rest benefits cardiac structure and function post-myocardial infarction in mice.

	MI+Rest	MI
**Echocardiography (Baseline)**		
LVIDd (mm)	4.01±0.03	4.00±0.01
LVIDs (mm)	2.43±0.02	2.41±0.02
EF (%)	76.45±0.31	76.55±0.28
FS (%)	39.48±0.27	39.57±0.25
HR (bpm)	458±12	438±7
**Echocardiography (1 week)**		
LVIDd (mm)	4.91±0.06	4.91±0.07
LVIDs (mm)	3.53±0.06	3.63±0.08
EF (%)	61.05±0.87*	57.84±1.04
FS (%)	28.23±0.55*	26.22±0.64
HR (bpm)	458±9	460±9
**Echocardiography (4 weeks)**		
LVIDd (mm)	5.15±0.07	5.23±0.08
LVIDs (mm)	3.75±0.07	3.95±0.09
EF (%)	59.24±0.77*	55.04±1.14
FS (%)	27.12±0.48*	24.61±0.65
HR (bpm)	473±12	474±6
**Echocardiography (8 weeks)**		
LVIDd (mm)	5.18±0.07*	5.42±0.08
LVIDs (mm)	3.84±0.06*	4.18±0.09
EF (%)	57.46±0.52**	52.23±1.10
FS (%)	26.00±0.31**	23.00±0.61
HR (bpm)	471±12	450±9
**Pressure-volume hemodynamics (8 weeks)**		
SBP (mmHg)	92.20±0.51*	89.74±0.95
DBP (mmHg)	62.30±1.27	59.58±1.00
MAP (mmHg)	71.54±0.87*	68.93±0.79
LVESP (mmHg)	94.12±0.78*	89.60±1.02
LVEDP (mmHg)	0.58±0.98	3.11±1.71
LVESV (µl)	25.50±1.50*	38.23±2.82
LVEDV (µl)	48.91±1.56*	57.38±2.62
SV (µl)	23.41±0.99*	19.14±0.53
CO (mL/min)	11.73±0.37*	9.86±0.29
dP/dt_{max} (mmHg/sec)	7346±350*	6162±244
dP/dt_{min} (mmHg/sec)	-6885±296*	-5542±351
ESPVR slope (mmHg/µl)	1.83±0.25*	0.98±0.09
HR (bpm)	507±23	517±15

LVIDd, left ventricle internal dimensions at diastole; LVIDs, LV internal dimensions at systole; % EF, % ejection fraction; % FS, % fractional shortening; HR, heart rate; SBP, systolic blood pressure; DBP, diastolic blood pressure; MAP, mean arterial pressure; LVESP, left ventricular end systolic pressure; LVEDP, left ventricular end diastolic pressure; LVESV, left ventricular end systolic volume; LVEDV, left ventricular end diastolic volume; SV, stroke volume; CO, cardiac output; dP/dt_{max} and dP/dt_{min}, maximum and minimum first derivative of LV pressure; ESPVR, end systolic pressure-volume relationship; HW, heart weight; HW:BW, HW:body weight ratio; HW:TL, HW:tibia length ratio. *$P<0.05$, **$P<0.001$ MI control vs. MI+Rest, by unpaired Student's *t*-test. n=10 mice/group for echocardiography, n=9 mice/group for hemodynamics and morphometry. Data are mean ± s.e.m.

Supplemental Table 3. Rest in *Clock*$^{\Delta 19/19}$ mice with heart disease.

	***Clock*$^{\Delta 19/19}$ TAC+Rest**	***Clock*$^{\Delta 19/19}$ TAC**	***Clock*$^{\Delta 19/19}$ Sham+Rest**	***Clock*$^{\Delta 19/19}$ Sham**
**Echocardiography (Baseline)**				
LVIDd (mm)	3.90±0.02	3.92±0.02	3.96±0.04	3.95±0.02
LVIDs (mm)	2.31±0.01	2.32±0.01	2.36±0.01	2.35±0.01
EF (%)	78.05±0.14	77.97±0.08	77.61±0.26	77.69±0.27
FS (%)	40.86±0.13	40.78±0.08	40.48±0.25	40.54±0.24
HR (bpm)	450±4	440±4	469±10	457±8
**Echocardiography (4 weeks)**				
LVIDd (mm)	4.14±0.03*	4.40±0.07	4.01±0.03	4.04±0.03
LVIDs (mm)	2.69±0.05*	3.03±0.08	2.39±0.02	2.41±0.02
EF (%)	71.02±0.94*	65.69±1.00	77.50±0.11	77.51±0.08
FS (%)	35.05±0.74*	31.21±0.67	40.38±0.10	40.39±0.06
IVSd (mm)	0.80±0.01*	0.83±0.01	0.63±0.01	0.63±0.01
LVPWd (mm)	0.79±0.01*	0.82±0.01	0.63±0.01	0.63±0.01
HR (bpm)	456±5	453±7	457±9	459±5
**Morphometry (4 weeks)**				
HW (mg)	192.00±8.21	217.63±12.13	126.00±1.34	128.80±1.24
HW:BW (mg/g)	6.81±0.24*	7.99±0.40	4.50±0.06	4.35±0.06
HW:TL (mg/mm)	9.24±0.39	10.44±0.54	6.12±0.04	6.18±0.05

LVIDd, left ventricle internal dimensions at diastole; LVIDs, LV internal dimensions at systole; % EF, % ejection fraction; % FS, % fractional shortening; HR, heart rate; IVSd, interventricular septal wall thickness at diastole; LVPWd, left ventricular posterior wall thickness at diastole; HW, heart weight; HW:BW, HW:body weight ratio; HW:TL, HW:tibia length ratio. *$P<0.05$ *Clock*$^{\Delta 19/19}$ TAC vs. *Clock*$^{\Delta 19/19}$ TAC+Rest, by unpaired Student's *t*-test. n=5 mice/group for sham, n=7-9 mice/group for TAC. Data are mean ± s.e.m.

Supplemental Table 4. Rest gene cassette.

Transcript ID	Gene Symbol	Gene Description	Rest	Control	Fold Change (Rest vs. Control)	Entrez Gene ID
17548055	BC023105		0.92	-0.50	2.66	667597
17296489	Nnt	Nicotinamide nucleotide transhydrogenase	0.18	-0.97	2.22	18115
17354589	Gm4841	Predicted gene 4841	0.75	-0.25	2.00	225594
17481960	Arntl	Aryl hydrocarbon receptor nuclear translocator-like	0.45	-0.47	1.90	11865
17350932	BC023105		0.63	-0.26	1.86	667597
17421540	Nppb	Natriuretic peptide type B	0.33	-0.55	1.84	18158
17212087	Npas2	Neuronal PAS domain protein 2	0.39	-0.43	1.77	18143
17324835	Tfrc	Transferrin receptor	0.41	-0.36	1.71	22042
17424088	Mir5123	microRNA 5123	0.33	-0.38	1.64	100628614
17464061	Gm10400	Predicted gene 10400	0.33	-0.33	1.58	100093700
17548256			0.09	-0.54	1.54	
17457318	Mir490	microRNA 490	0.20	-0.41	1.53	735279
17350921	F830016B08 Rik	RIKENcDNAF830016B08 gene	0.51	-0.08	1.51	240328
17424092	Gm25931	Predicted gene, 25931 [Source: MGI Symbol; Acc: MGI: 5455708]	0.35	-0.24	1.51	
17281084	Egln3	egl-9 family hypoxia-inducible factor 3	0.30	-0.28	1.49	112407
17332228	Rcan1	Regulator of calcineurin 1	0.27	-0.30	1.49	54720
17438963	Ppbp	pro-platelet basic protein	0.42	-0.14	1.47	57349
17249980	Igtp	Interferon gamma induced GTPase	0.35	-0.20	1.47	16145
17385654	Itgb6	Integrin beta 6	0.23	-0.28	1.42	16420
17376549	Prnd	Prion protein dublet | prion protein gene complex	0.19	-0.29	1.40	26434 / 111368
17510040	Crtc1	CREB regulated transcription coactivator 1	0.24	-0.24	1.39	382056
17222193	Ankrd23	Ankyrin repeat domain 23	0.33	-0.14	1.39	78321
17450501	Gbp10	guanylate-binding protein 10	0.25	-0.22	1.38	626578
17392722			0.38	-0.09	1.38	
17550156			0.38	-0.09	1.38	
17381509	5031426D15 Rik	RIKENcDNA5031426D15 gene	0.17	-0.29	1.38	68144
17261107	Rel	Reticuloendotheliosis oncogene	0.18	-0.28	1.38	19696
17266563	Wsb1	WD repeat and SOCS box-containing 1	0.28	-0.18	1.37	78889

17534892	Zfp449	Zinc finger protein 449	0.25	-0.21	1.37	78619
17280817	Scin	scinderin	0.27	-0.18	1.37	20259
17520073	Nt5e	5'-nucleotidase, ecto	0.22	-0.22	1.36	23959
17548472			0.15	-0.29	1.36	
17474932			0.32	-0.11	1.35	
17248754	Adam19	A disintegrin and metallopeptidase domain 19 (meltrin beta)	0.19	-0.24	1.34	11492
17341540	Tnfrsf12a	Tumor necrosis factor receptor superfamily, member 12a	0.20	-0.23	1.34	27279
17227910	Hmcn1	Hemicentin 1	0.19	-0.23	1.34	545370
17456934	Mest	Mesoderm specific transcript	0.25	-0.16	1.33	17294
17229307	Dusp27	Dual specificity phosphatase 27 (putative)	0.18	-0.23	1.33	240892
17232235	Ctgf	Connective tissue growth factor	0.15	-0.25	1.32	14219
17438189	Rasl11b	RAS-like, family 11, member B	0.22	-0.18	1.32	68939
17550246			0.21	-0.19	1.32	
17245433	Msrb3	Methionine sulfoxide reductase B3	0.18	-0.22	1.32	320183
17472598	C2cd5	C2 calcium-dependent domain containing 5	0.25	-0.15	1.32	74741
17317307	Gm24041	Predicted gene, 24041 [Source: MGI Symbol; Acc: MGI: 5453818]	0.13	-0.27	1.32	
17490149	Cd33	CD33 antigen	0.14	-0.26	1.32	12489
17426784	2310067E19 Rik	RIKENcDNA2310067E19 gene	0.18	-0.22	1.32	76455
17267632	Dgke	Diacylglycerol kinase, epsilon	0.20	-0.19	1.31	56077
17315686	C7	Complement component 7	0.11	-0.28	1.31	109828
17267420	Ypel2	yippee-like 2 (Drosophila)	0.17	-0.22	1.30	77864
17238934	Syne1	Spectrin repeat containing, nuclear envelope 1	0.07	-0.31	1.30	64009
17381515	Gm10115	Predicted gene 10115 [Source: MGI Symbol; Acc: MGI: 3641675]	0.12	-0.26	1.30	
17302588	Gm23437	Predicted gene, 23437 [Source: MGI Symbol; Acc: MGI: 5453214]	-0.32	0.05	-1.30	
17364986	Gm24400	Predicted gene, 24400 [Source: MGI Symbol; Acc: MGI: 5454177]	-0.22	0.16	-1.30	

17339987	Gm24240	Predicted gene, 24240 [Source: MGI Symbol; Acc: MGI: 5454017]	-0.18	0.21	-1.31	
17326944	LOC102637417 |Gm7735	keratin-associated protein 20-2-like | predicted gene 7735 [Source: MGI Symbol; Acc: MGI: 3649168]	-0.28	0.10	-1.31	102637417
17532629	mt-Ts2| mt-Tl2	Mitochondrially encoded tRNA serine 2 [Source: MGI Symbol; Acc: MGI:102474] | mitochondrially encoded tRNA leucine 2 [Source: MGI Symbol; Acc: MGI: 102481]	-0.02	0.36	-1.31	
17268884	Nr1d1	Nuclear receptor subfamily 1, group D, member 1	-0.13	0.26	-1.31	217166
17366918	Mir466d	microRNA 466d	-0.14	0.25	-1.32	100124465
17475777	Fbl	fibrillarin	-0.17	0.23	-1.32	14113
17532593	mt-Tf	Mitochondrially encoded tRNA phenylalanine [Source: MGI Symbol; Acc: MGI: 102487]	-0.28	0.14	-1.33	
17550382			-0.18	0.24	-1.34	
17532489			-0.21	0.22	-1.35	
17303625	Nr1d2	Nuclear receptor subfamily 1, group D, member 2	-0.27	0.18	-1.36	353187
17407188	Gm24046	Predicted gene, 24046 [Source: MGI Symbol; Acc: MGI: 5453823]	-0.10	0.36	-1.37	
17364098	Acta2	Actin, alpha 2, smooth muscle, aorta	-0.17	0.30	-1.38	11475
17340673	Fndc1	Fibronectin type III domain containing 1	-0.24	0.22	-1.38	68655
17472760	Bhlhe41	Basic helix-loop-helix family, member e41	-0.20	0.27	-1.38	79362
17280479	Gm25865	Predicted gene, 25865 [Source: MGI Symbol; Acc: MGI: 5455642]	-0.24	0.23	-1.39	
17550004			-0.24	0.23	-1.39	
17251527	Per1	Period circadian clock 1	-0.26	0.21	-1.39	18626
17548916			-0.29	0.21	-1.42	
17511693	Ces1d	Carboxylesterase 1D	-0.30	0.21	-1.43	104158
17503937	Mt2	Metallothionein 2	-0.17	0.37	-1.45	17750

17461414	Bhlhe40	Basic helix-loop-helix family, member e40	-0.18	0.36	-1.46	20893
17255987	Tcap	titin-cap	-0.28	0.28	-1.47	21393
17464654	Pdk4	Pyruvate dehydrogenase kinase, isoenzyme 4	-0.27	0.37	-1.56	27273
17513995	Acta1	actin, alpha 1, skeletal muscle	-0.36	0.28	-1.56	11459
17212874	Coq10b	Coenzyme Q10 homolog B (S. cerevisiae)	-0.35	0.30	-1.57	67876
17400862	Hmgcs2	3-hydroxy-3-methylglutaryl-Coenzyme A synthase 2	-0.40	0.26	-1.58	15360
17219206	Gm26110	Predicted gene, 26110 [Source: MGI Symbol; Acc: MGI: 5455887]	-0.12	0.56	-1.61	
17400568	Rnu1b6	U1b6 small nuclear RNA	-0.12	0.56	-1.61	19847
17400579	Rnu1b1| Rnu1b2	U1b1 small nuclear RNA | U1b2 small nuclear RNA	-0.12	0.56	-1.61	19844 / 19845
17400588	Rnu1b6	U1b6 small nuclear RNA	-0.12	0.56	-1.61	19847
17400590	Gm25890| Gm22614	Predicted gene, 25890 [Source: MGI Symbol; Acc: MGI: 5455667] | predicted gene, 22614 [Source: MGI Symbol; Acc: MGI: 5452391]	-0.12	0.56	-1.61	
17408038	Rnu1b1| Rnu1b2	U1b1 small nuclear RNA | U1b2 small nuclear RNA	-0.12	0.56	-1.61	19844 / 19845
17408063	Rnu1b6	U1b6 small nuclear RNA	-0.12	0.56	-1.61	19847
17408065	Gm25890| Gm22614	Predicted gene, 25890 [Source: MGI Symbol; Acc: MGI: 5455667] | predicted gene, 22614 [Source: MGI Symbol; Acc: MGI: 5452391]	-0.12	0.56	-1.61	
17425278	Gm22042	predictedgene,22042 [Source: MGI Symbol; Acc: MGI: 5451819]	-0.12	0.56	-1.61	
17480729	Ucp3	Uncoupling protein 3 (mitochondrial, proton carrier)	-0.31	0.39	-1.61	22229
17520288	Gm22866	predictedgene,22866 [Source: MGI Symbol; Acc: MGI: 5452643]	-0.18	0.63	-1.75	
17477979	Dbp	D site albumin promoter binding protein	-0.52	0.57	-2.12	13170

Microarray data from hearts collected at ZT07, n=3 hearts/group. Data show the mean normalized expression and mean fold change between groups.

Supplemental Table 5. GEO datasets of human myocardial tissue gene expression.

GEO Accession	Patient Cohort	Tissue	Microarray Platform	Ref.
GSE1145	Aortic stenosis and LV hypertrophy (n=7)<br>Control (coronary artery disease with normal LV function) (n=4)	LV biopsy	Affymetrix Human Genome U95 Version 2 Array	-
GSE10161	Aortic stenosis (n=20)<br>Control (n=7)	LV biopsy	Affymetrix Human Genome U133A Array	Petretto et al. *Nat Genet*. 2008;40(5):546-52
GSE3585	Dilated cardiomyopathy (n=7)<br>Non-failing control (n=5)	LV biopsy	Affymetrix Human Genome U133A Array	Barth et al. *J Am Coll Cardiol*. 2006;48(8):1610-7
GSE42955	Dilated cardiomyopathy (n=12)<br>Ischemic cardiomyopathy (n=12)<br>Control (n=5)	LV biopsy	Affymetrix Human Gene 1.0 ST Array	Molina-Navarro et al. *PLoS One*. 2013;8(12):e79792
GSE79962	Dilated cardiomyopathy (n=9)<br>Ischemic heart disease (n=11)<br>Non-failing donors (n=11)	LV biopsy	Affymetrix Human Gene 1.0 ST Array	Matkovich et al. *Crit Care Med*. 2017;45(3):407-414
GSE16499	Ischemic heart failure (n=15)<br>Non-failing control (n=15)	LV biopsy	Affymetrix Human Exon 1.0 ST Array	Kong et al. *Circ Cardiovasc Genet*. 2010;3(2):138-46

Supplemental Table 6. Commonly prescribed cardiac medications listed by the American Heart Association target rest-responsive genes in the heart.

Drug Class	Drug Name (generic)	Major Trade Name	Rhythmic Genes (Murine heart, CircaDB)	Rest-responsive Genes (Rest model, murine heart microarrays)
Anticoagulants	Apixaban	Eliquis	*Abcb1a, Abcg2*	*Abcb1a*
	Dabigatran	Pradaxa	*Abcb1a, Ces1*	*Abcb1a, Ces1*
	Heparin	various	*Ccl5, Fgfr1, Fgfr4, Hgf, Vegfa*	*Fgfr4, Hgf, Vegfa*
	Rivaroxaban	Xarelto	*Abcb1a, Abcg2*	*Abcb1a*
	Warfarin	Coumadin	*Cyp1a1*	-
Antiplatelet Agents	Aspirin		*Abcb1a, Ccna2, Ednra, Hsp5a, Mapk6, Nfkb1, Nfkbia, Prkaa1, Prkab1, Ptgs1, Rps6ka3*	*Abcb1a, Ccna2, Ccnd1, Hspa5, Mapk6, Prkaa2*
	Clopidogrel	Plavix	*Abcb1a, Ces1*	*Abcb1a, Ces1, Slc22a2*
	Dipyridamole		*Abcb1a, Abcc5, Pde4a, Rcan1, Slco2b1*	*Abcb11, Abcb1a, Abcc5, Rcan1*
	Prasugrel	Effient	-	-
	Ticagrelor	Brilinta	*Abcb1a*	*Abcb1a*
ACE Inhibitors	Benazepril	Lotensin	*Ace, Mthfr*	-
	Captopril	Capoten	*Abcb1a, Ace*	*Abcb1a, Lta4h*
	Enalapril	Vasotec	*Abcb1a, Ace*	*Abcb1a*
	Fosinoprill	Monopril	*Ace*	-
	Lisinopril	Prinivil/Zestril	*Ace*	-
	Moexipril	Univasc	*Ace, Ace2*	-
	Perindopril	Aceon	*Ace*	-
	Quinapril	Accupril	*Ace*	-
	Ramipril	Altace	*Ace*	-
	Trandolapril	Mavik	*Ace, Ces1*	*Ces1*
Angiotensin II Receptor Blockers	Candesartan	Atacand	*Abcb1a, Agtr1a, Ptgs1*	*Abcb1a*
	Eprosartan	Teveten	*Agtr1a*	-
	Irbesartan	Avapro	*Agtr1a, Jun, Ptgs1*	-
	Losartan	Cozaar	*Abcb1a, Agtr1a*	*Abcb11, Abcb1a*
	Telmisartan	Micardis	*Abcb1a, Abcg2, Agtr1a*	*Abcb11, Abcb1a*
	Valsartan	Diovan	*Agtr1a*	-
Angiotensin-Receptor Neprilysin Inhibitors	Sacubitril/valsartan	Entresto	-	-
Beta Blockers	Acebutolol	Sectral	*Abcb1a, Adrb1*	*Abcb1a*
	Atenolol	Tenormin	*Adrb1*	*Abcb11*
	Betaxolol	Kerlone	*Adrb1*	-
	Bisoprolol/ hydrochlorothiazide	Ziac	*Abcb1a, Adrb1*	*Abcb1a*
	Bisoprolol	Zebeta	*Abcb1a, Adrb1*	*Abcb1a*
	Metoprolol	Lopressor/Toprol XL	*Abcb1a, Adrb1*	*Abcb1a, Slc22a2*
	Nadolol	Corgard	*Abcb1a, Adrb1*	*Abcb1a*
	Propranolol	Inderal	*Abcb1a, Adrb1, Cyp1a1*	*Abcb1a, Slc22a2*
	Sotalol	Betapace	*Adrb1, Kcnh2*	-

Combined Alpha and Beta Blockers	Carvedilol	Coreg	*Abcb1a, Adra1a, Adra1d, Adrb1, Cyp1a1, Gja1, Hif1a, Kcnh2, Ndufc2, Nppb, Ptgs1, Vcam1, Vegfa, Xdh*	*Abcb1a, Gja1, Hif1a, Nppb, Vcam1, Vegfa*
	Labetalol hydrochloride	Normodyne/ Trandate	*Adra1a, Adra1d, Adrb1*	-
Calcium Channel Blockers	Amlodipine	Norvasc/Lotrel	*Abcb1a, Cyp1a1, Nos2*	*Abcb1a, Cacna1c*
	Diltiazem	Cardizem/Tiazac	*Abcb1a*	*Abcb1a, Cacna1c*
	Felodipine	Plendil	*Abcb1a, Cacnb2, Calm2, Calm3, Nr3c2, Pde1b*	*Abcb11, Abcb1a, Cacna1c, Tnnc2*
	Nifedipine	Adalat/Procardia	*Abcb1a, Cacnb2, Calm2, Calm3, Cyp1a1*	*Abcb11, Abcb1a, Cacna1c*
	Nimodipine	Nimotop	*Cacnb2, Cacnb3, Cacnb4, Nr3c2*	*Cacna1c, Cacnb3, Cacnb4*
	Nisoldipine	Sular	*Abcb1a, Cacnb2*	*Abcb1a, Cacna1c*
	Verapamil	Calan/Verelan	*Abcb1a, Abcc1, Abcg2, Adra1a, Adra1d, Cacnb2, Cacnb3, Cacnb4, Kcnh2, Scn5a*	*Abcb11, Abcb1a, Cacna1c, Cacnb3, Cacnb4, Slc22a5*
Cholesterol-lowering Medications	Atorvastatin	Lipitor	*Abcb1a, Abcc1, Abcc5, Slco2b1*	*Abcb11, Abcb1a, Abcc5, Dpp4*
	Digitoxin		*Abcb1a*	*Abcb1a, Cyp11a1*
	Digoxin	Lanoxin	*Abcb1a*	*Abcb11, Abcb1a, Cyp11a1*
	Ezetimibe/ Simvastatin	Vytorin	*Abcb1a, Abcg2*	*Abcb11, Abcb1a*
	Lovastatin	Advicor	*Abcb1a*	*Abcb11, Abcb1a*
	Rosuvastatin	Crestor	*Abcc1, Abcg2, Slco2b1*	*Abcb11, Slc7a11*
	Simvastatin	Zocor	*Abcb1a*	*Abcb11, Abcb1a*
Diuretics	Amiloride	Midamor	*Scnn1g*	*Scnn1a, Slc22a2*
	Bumetanide	Bumex	*Slc12a4*	-
	Chlorothiazide	Diuril	-	-
	Chlorthalidone	Hygroton	-	-
	Furosemide	Lasix	*Pgd, Slco2a1*	*Slc22a5*
	Hydro-chlorothiazide	Esidrix/Hydrodiuril	-	-
	Indapamide	Lozol	-	-
	Spironolactone	Aldactone	*Abcb1a, Nr3c1, Nr3c2*	*Abcb11, Abcb1a, Cacna1c, Srd5a1*
Vasodilators	Hydralazine	Apresoline	*P4ha1*	*P4ha1*
	Isosorbide dinitrate	Isordil	-	-
	Minoxidil	Loniten	*Ptgs1*	*Ren1*
	Nesiritide	Natrecor	*Npr2, Npr3*	-
	Nitroglycerin	Nitrostat	-	-
	Nitroprusside	Nitropress	*Cyp1a1*	-
